# Implementation readiness of artificial intelligence in *in vitro* fertilization: a multi-framework appraisal with special reference to advanced reproductive age patients

**DOI:** 10.3389/fendo.2026.1910448

**Published:** 2026-09-07

**Authors:** Chia Lin Chang

**Affiliations:** 1 Department of Obstetrics and Gynecology, Chang Gung Memorial Hospital, Linkou Medical Center, Taoyuan, Taiwan; 2 Chang Gung University College of Medicine, Taoyuan, Taiwan

**Keywords:** advanced maternal age, artificial intelligence, clinical decision support, embryo selection, implementation science, *in vitro* fertilization, technology readiness level

## Abstract

Artificial intelligence (AI) has advanced rapidly across medicine, and IVF—with its image-rich laboratory workflows, structured cycle data, and measurable endpoints—is among the most amenable fields for integration. Three applications—outcome prediction (T1), stimulation dosing (T2), and radio-frequency identification (RFID) quality tracking (T3)—require no new capital hardware and are immediately deployment-ready, and as professional adoption rose from 24.8% (2022) to 53.2% (2025), the field reached a stratified state of readiness. AI in IVF presents three implementation-relevant findings: the highest-profile technology (embryo selection) is not the most deployment-ready; the lowest-profile (outcome prediction) has the greatest near-term impact; and patients ≥40 years show the largest AI discriminatory advantage. Nine AI technologies (T1–T9) were evaluated across the IVF clinical workflow using four implementation frameworks (TRL/MLTRL, IDEAL, Rogers’ Diffusion of Innovation, NASSS) with OCEBM evidence grading, organized by deployment readiness to derive a deployable toolkit for the 2026 clinic and a synthesis for advanced reproductive age (ARA) patients. Three deployment waves emerged. Wave 1 (≤5 years) comprises outcome prediction (T1), stimulation dosing (T2), and RFID quality tracking (T3), immediately deployable as decision-support adjuncts (12–18 months) pending prospective RCT validation. Wave 2 (5–10 years) comprises AI ultrasound monitoring (T4), sperm analysis and selection (T5), embryo assessment (T6), and endometrial receptivity (T7), positioned for pilot-stage deployment with FDA-cleared FOLLISCAN (T4) leading. Wave 3 (beyond 2031)—miscarriage prediction (T8) and robotic ICSI (T9)—comprises horizon technologies contingent on ethical and engineering resolution. Embryo-selection AUC rises with maternal age from 0.596 (<35 years) to 0.768 (≥43 years; 29% relative increase), positioning ARA patients as candidates for greater AI value pending prospective multicenter validation, while platform-specific findings from the two largest embryo-selection RCTs (LOTUS met endpoint, n=442; iDAScore failed non-inferiority, n=1,066) indicate embryo assessment must be evaluated by platform, not as a uniform category. The three-wave framework lets clinic directors sequence AI investment by readiness; for Wave 1, procurement—not validation—is rate-limiting, and four prerequisite clusters (multicenter RCT completion, regulatory harmonization, ethical frameworks for AI-informed transfer, and workforce/IT infrastructure) must advance in parallel. ARA patients constitute the primary stress test, and ultimately success depends not on algorithmic sophistication but on rigorous validation, governance, and equitable deployment.

## Graphical Abstract

**Figure:**
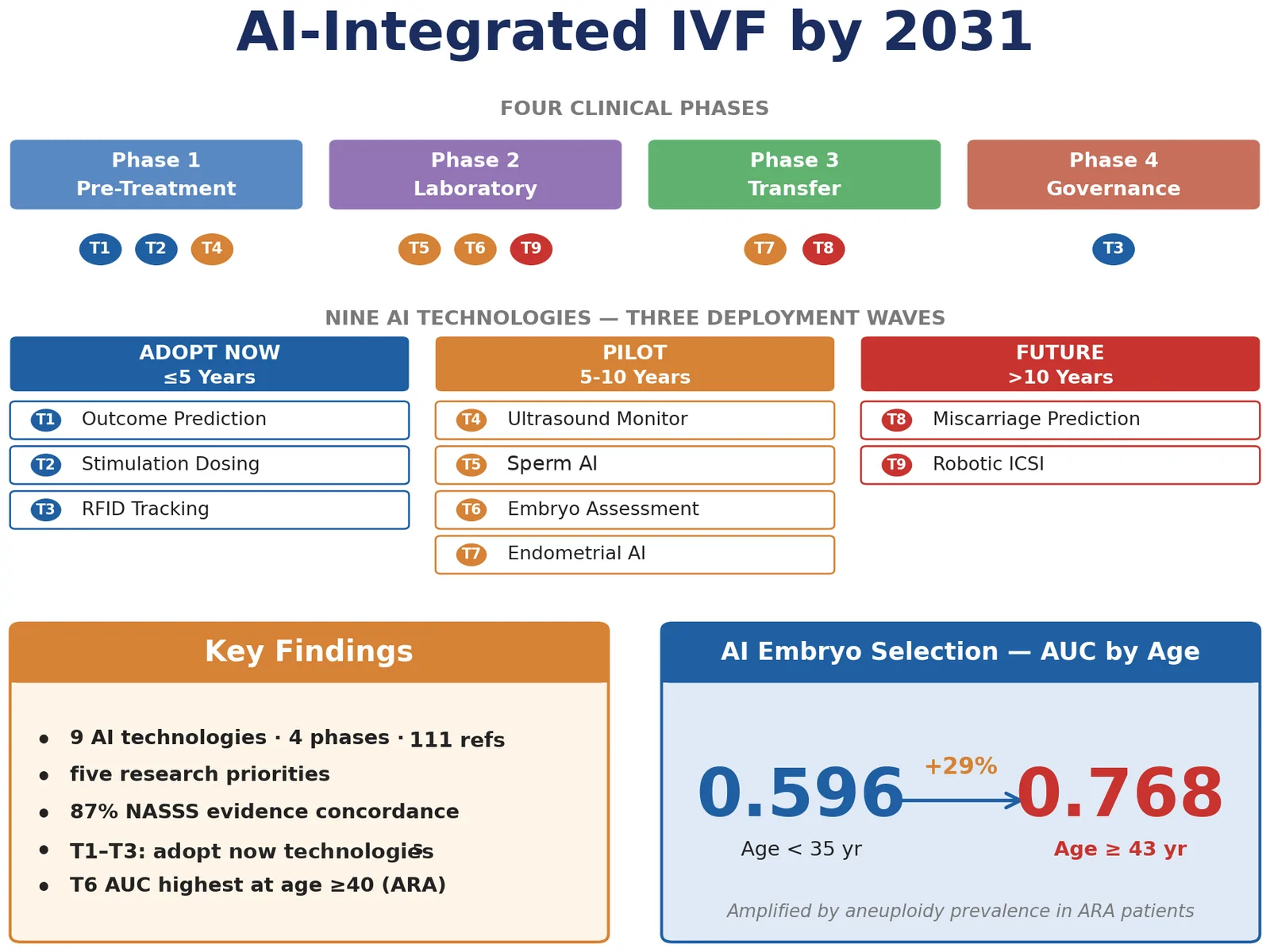
Three-tier visual summary of an AI-integrated IVF strategy by 2031. Top tier: four clinical phases of the IVF protocol (Phase 1 Pre-Treatment, Phase 2 Laboratory, Phase 3 Transfer, Phase 4 Governance) with T-number icon assignments for each phase. Middle tier: nine AI technologies (T1–T9) organized into three deployment waves — Adopt Now (≤5 yr; T1 Outcome Prediction, T2 Stimulation Dosing, T3 RFID Tracking), Pilot (5–10 yr; T4 Ultrasound Monitor, T5 Sperm AI, T6 Embryo Assessment, T7 Endometrial AI), and Future (>10 yr; T8 Miscarriage Prediction, T9 Robotic ICSI) — with individual technology cards color-coded by wave (blue, amber, red, respectively). Bottom tier: left panel — five key findings: 9 AI technologies across 4 phases; five research priorities; 87% NASSS evidence concordance; three Tier 1 adopt-now technologies (T1–T3); and T6 AUC increases with maternal age (ARA amplification). Right panel — AI embryo selection discriminatory performance across five maternal age strata (1): AUC 0.596 (age <35 yr), 0.640 (35–37 yr), 0.646 (38–40 yr), 0.679 (41–42 yr), and 0.768 (age ≥43 yr; +29% relative increase versus age <35 yr); the progressive AUC rise is amplified by the increasing aneuploidy prevalence in ARA patients (2).

## Highlights

- This review evaluates nine AI tools and asks which are ready for use today, which are still being tested, and which are still years away. We identify three tools ready for use in the next 12–18 months: software that helps doctors give patients more accurate estimates of their chance of having a baby through IVF, software that helps tailor the hormone dose for each patient during stimulation, and an electronic safety system that prevents mix-ups in the laboratory. None of these requires expensive new equipment. AI tools that help select the embryo most likely to lead to pregnancy show promise but have produced mixed results in two large clinical trials. So they should be treated as helpers — not replacements — for embryologist judgment. AI tools that predict miscarriage or that perform robotic injection of sperm into the egg are still in early research and not ready for routine use. For patients aged 40 and over, who face the greatest difficulties with IVF success, AI could offer proportionally greater benefit for identifying which embryos and which treatment plans are most likely to succeed. The review uses a recurring case — a 41-year-old patient with diminished ovarian reserve — to show how each AI tool would be used at each stage of her treatment journey. Importantly, four developments must occur between now and 2031 to make the AI-integrated IVF clinic a reality. They include clinical trials with live-birth outcomes, harmonized regulations across countries, ethical frameworks for AI-informed decisions, and training for doctors and embryologists/integration of AI with hospital electronic systems.

## Introduction

In the last five years, artificial intelligence has moved from experimental curiosity to clinical deployment across radiology, pathology, ophthalmology, and cardiology. Reproductive medicine — and *in vitro* fertilization (IVF) in particular — occupies a distinctive position in this landscape. At every clinical stage, IVF generates multimodal data (i.e., demographics, hormonal profiles, ultrasound imaging, time-lapse embryo video, genetic testing, and cycle outcome records) that are captured in a closed-loop workflow with a measurable endpoint (live birth). This data architecture makes it one of the most AI-amenable disciplines in medicine (3–5).

Since 1978, IVF has produced over twelve million births (6, 7), yet only about one-third of cycles result in live birth (7, 8). This establishes a clear performance ceiling that AI-augmented decision-making could help address. Importantly, the first FDA-cleared Machine Learning (ML) embryo assessment tool received 510(k) clearance in September 2025, and the first live birth from remotely controlled intracytoplasmic sperm injection (ICSI; operators separated by 3,700 km) was reported in 2025 (9). In addition, two completed multicenter RCTs of AI embryo selection have reported results. Key milestones that were achieved between approximately 2021 and 2026 are summarized in **Supplementary Table 1**.

Global AI adoption among IVF professionals rose from 24.8% in 2022 to 53.2% (any AI use across clinical domains) in 2025, with 83.62% of 2025 respondents indicating likely investment in AI within 1–5 years (10). However, these figures conceal a wide variance: different technologies are at vastly different readiness stages, with different evidence bases, different barriers to deployment, and different patient impacts. As such, the field is shifting from asking whether AI can improve outcomes toward asking which tools are ready for deployment, even as the former question remains incompletely answered for most technologies. The peer-reviewed literature primarily documents what AI can do under controlled conditions, but provides limited guidance on deployment sequence, resource requirements, or organizational prerequisites — creating decision paralysis for clinical leaders evaluating AI integration in IVF.

Indeed, AI in IVF presents three counterintuitive realities that complicate implementation decisions. First, the technology with the highest commercial profile and research investment — AI embryo selection — is not the most ready for definitive deployment. The two large randomized trials have yielded divergent results. Second, the technology with a comparatively low profile — AI outcome prediction — could have the strongest near-term cost-avoidance case among the nine technologies considered (i.e., T1–T9). It requires no hardware capital and addresses prognosis miscalibration that affects every IVF patient. Third, the patient population most often dismissed as low-yield — patients aged ≥40 — is the population in whom AI’s discriminatory advantage is greatest. AI embryo selection demonstrates a steep, age-dependent increase in discriminatory accuracy, yielding its highest predictive value in patients over 40 (1). These three counterintuitive realities structure the analysis that follows.

The review opens with a Methodology chapter establishing the analytical framework (four implementation frameworks, OCEBM evidence grading, NASSS sensitivity analysis, and conflict-of-interest protocol). It then presents the deployable 2026 AI toolkit early, before the full evidence appraisal, so that decision-makers can extract actionable guidance without first reading the technology-by-technology assessment. The Technology Assessment section organizes the nine technologies by their three-wave deployment readiness (**Supplementary Table 2**), and a phase assignment of these technologies within the IVF clinical protocol is presented in **Supplementary Table 3** as a secondary classification. The Patient Perspective section closes the technology-evidence block with the patient perspective on AI deployment. Subsequent chapters provide the evidence quality and critical appraisal (Evidence Quality and Critical Appraisal, encompassing study quality, research priorities, deployment-timeline confidence, and resource impact), failure modes (AI Failure Modes), implementation prerequisites (Implementation Prerequisites), procurement and integration considerations (Procurement), and an ARA-specific clinical synthesis built around a recurring patient case (The ARA Patient: Integrated Clinical Synthesis).

Among the nine AI technologies, AI embryo selection (T6) has been identified as the primary anticipated benefit of AI in ART (10). However, four converging factors — divergent randomized controlled trial (RCT) results, platform-specific performance sensitivity, time-lapse hardware dependency, and the absence of a live-birth-endpoint regulatory clearance — have collectively placed AI embryo selection at the 5–10-year horizon, behind three immediately deployable software-only technologies (T1, T2, T3) as decision-support adjuncts. These results underscore that cautious optimism, rather than unconditional enthusiasm, is warranted.

While the projected trajectory is optimistic, the evolutionary path of AI in the last five years — from concept to FDA clearance for embryo selection AI, from zero to robotic ICSI clinical deployment — beginning with 2 live births from the first automated ICSIA pilot (11), then the first fully remote ICSI live birth (9), then a Day 0 proof-of-concept multi-system series (12), and company-reported expansion to approximately 20 cumulative live births across all Conceivable systems by late 2025, from no RCTs to two completed multicenter trials — provides compelling empirical evidence that the software-based AI projections in this review are achievable within a 5-year horizon.

While these projections apply broadly, their clinical impact is not uniformly distributed across patient populations. For patients of advanced reproductive age (ARA; ≥40 years), the readiness stratification carries direct clinical implications. The three software-only technologies in Wave 1 — AI outcome prediction (T1), stimulation dosing optimization (T2), and radio-frequency identification (RFID) quality tracking (T3) —are immediately applicable to ARA patients as decision-support adjuncts, where accurate prognosis and individualized dosing carry the greatest per-cycle value. Among the Wave 2 technologies, AI embryo selection (T6) demonstrates amplified discriminatory accuracy with advancing maternal age, making it the highest-priority medium-term investment for ARA-focused programs. Hardware-dependent technologies (T9) and those requiring multi-stream validation (T8) are projected beyond the 2031 horizon, but their eventual deployment will proportionally benefit older patients who carry the highest risk of failed implantation and early pregnancy loss.

## Methodology

This review applies a multi-framework approach combining Technology Readiness Level (TRL/MLTRL) scales, the IDEAL framework, Rogers’ Diffusion of Innovation theory, and the NASSS framework, with evidence graded using an OCEBM-adapted hierarchy. The nine candidate technologies (T1–T9) span the full IVF protocol and are analyzed for systematic AI benefit amplification in advanced reproductive age (ARA) patients. Coverage included PubMed/MEDLINE, Scopus, Google Scholar, and IEEE Xplore for English-language peer-reviewed articles, with a core search window of January 2021–March 2026 supplemented by foundational papers from January 2015 onward for technologies with pre-2021 origins (e.g., T3 RFID, T5 sperm-selection antecedents, T9 robotic ICSI), and by ASRM and ESHRE conference programs and FDA/EU MDR regulatory filings (**Supplementary Tables 1**, **4** [PRISMA-aligned]). Non-peer-reviewed sources were included only where they constituted the only available evidence and are classified at OCEBM Level 4–5 (**Supplementary Table 2**). The frameworks are complementary in scope: TRL/MLTRL for technical maturity (13–15); IDEAL/IDEAL-D for procedural staging (16, 17); Rogers for adoption trajectory (18); NASSS for organizational complexity (19); with DECIDE-AI informing early-stage clinical evaluation standards (20). TRL-level assignments were made by author judgment against the published rubrics for each framework, with full framework definitions, per-technology rationale, search strategy details, and NASSS sensitivity analysis methodology provided in the **Supplementary Methods**.

**Table 1** presents the NASSS domain complexity heatmap. RFID quality tracking is the only technology rated Simple overall; robotic ICSI and miscarriage prediction carry X (Complex) ratings in four or more domains. The Wider System domain rates Complicated or Complex for six of nine technologies, reflecting immature AI-specific regulatory frameworks (21). A structured sensitivity analysis re-examined each NASSS domain assignment against its supporting evidence: 55 of 63 ratings (87%) showed concordance with the underlying literature, and all discordant ratings, on adjudication, moved toward higher complexity (**Supplementary Methods**, Section S.M.5). As a narrative review, selection bias is inherent; a systematic conflict-of-interest screening was integrated into evidence evaluation, with studies in **Table 2** flagged for risk-of-bias when author–developer affiliations existed (**Supplementary Methods**).

**Table 1:** NASSS domain complexity assessment.

AI technology	Condition	Technology	Value prop.	Adopter system	Organization	Wider system	Embedding
	*Patient & procedure*	*Device & software*	*Costs & business model*	*Clinician & patient fit*	*Institutional capacity*	*Regulatory & economic env.*	*Long-term sustainability*
T1 AI Outcome Prediction	S	S	S	C	S	C	S
T2 AI Stimulation Dosing	S	S	C	C	S	S	C
T3 RFID Quality Tracking	S	S	S	S	C	S	S
T4 AI Ultrasound Monitoring	S	C	C	C	S	C	C
T5 Sperm AI	S	C	C	C	S	C	C
T6 AI Embryo Selection	S	C	C	C	C	S	C
T7 Endometrial Receptivity AI	S	C	C	C	S	C	C
T8 Miscarriage Prediction	C	C	X	X	C	X	X
T9 Robotic ICSI	C	X	C	X	X	X	X

Nine AI technologies rated across seven organizational domains using the NASSS framework (19). Cell shading encodes complexity: light blue, Simple (S); amber, Complicated (C); light red, Complex (X). Domain definitions: Condition, clinical context; Technology, algorithmic maturity; Value Prop., cost-effectiveness evidence; Adopter System, clinician acceptance; Organization, workflow integration; Wider System, regulatory environment; Embedding, long-term sustainability. Structured sensitivity analysis: 55/63 domain ratings (87%) showed concordance between the assigned complexity ratings and the supporting published evidence.

**Table 2:** Quality assessment of key clinical studies.

Technology domain	Study design	N	Primary endpoint	Key result	RoB	Key limitations
T1 Outcome Prediction — 22 (MLCS)	MC retrospective validation	4,635	LBP discrimination (PR-AUC, F1)	PR-AUC 0.75 vs SART 0.69 (p<0.05); reclassified 23%/11% to LBP ≥50%/≥75%	? Moderate	6 US centers; Univfy author affiliations (developer COI); retrospective; not yet prospectively tested for clinical impact
T2 Stimulation Dosing — 23	Multi-center retrospective (3 US ART centers)	18,591	MII oocytes (2PN, blastocysts)	Identifies per-patient dose–response curves; improves MII yield in low-prognosis patients	? Moderate	3 US centers; Alife Health employee co-authors (developer COI); no completed prospective RCT (IDoser NCT05948293 withdrawn)
T4 AI Ultrasound — CycleClarity (24)	Prosp. + hist.	291	MII oocytes; FSH	Not significant (NS) (+0.96 MII; −174 IU)	? Moderate	NS primary endpoint; commercial developer data; FOLLISCAN (MIM Fertility) also FDA 510(k) cleared with peer-reviewed multicenter validation (25)
T4 AI Ultrasound — 25 (FOLLISCAN)	Multicenter validation	1,689 (5,508 scans)	Follicle detection precision	98.2% precision; 93.3% F1 (≥10 mm)	○ Low	Multicenter (Poland, Argentina, Colombia, US); stable across ultrasound systems; annotation time reduced 2.5×
T5b Sperm Selection — 26	SC Blind Obs	102 cpls	Blastocyst rate	+10% blastocyst	? Moderate	Single center; surrogate endpoint; no live birth data
T6 Embryo Selection — 27	MC DB RCT	1,066	CPR (non-inferiority)	Failed NI	+ Low	Higher-than-expected control CPR; one TL platform; short FU
T6 Embryo Selection — 28 (LOTUS)	MC RCT	442	CPR (non-inferiority)	Met NI	– High (pre-peer-review)	Not powered for superiority; 7 US clinics only; static images
T6 Ploidy Prediction (Federated Learning) — 29	Retro cohort	10,065	AUC, ploidy	AUC 0.694–0.735 (5 hospitals)	**–** High	Retrospective; no prospective validation; regulatory unclear
T7 Endometrial Receptivity — 30	Retro (synthetic aug.)	Single center	Composite LBR	AUC 0.94	– High	Single-center; synthetic augmentation; no multicenter data
T8 Miscarriage Prediction — 31	MC retrospective	391	Miscarriage (Day-3)	AUC ~0.70	– High	Retrospective; AUC insufficient for clinical use
T9 Robotic ICSI — 12	PoC study	11 pts	Feasibility	5 LB/12 ET	– High	Very small N; overlapping author groups; no control arm

Study design, sample size, primary endpoint, key result, and risk of bias (RoB; encoded using Cochrane-style traffic light shading: + green = Low,? yellow = Moderate, − red = High) for representative studies across each technology domain.

## An AI toolkit for the 2026 IVF clinic

The Methodology section above establishes the analytical framework. This chapter translates that scaffolding into a deployable AI toolkit organized by clinical resource tier (**Table 3**). Subsequent chapters provide the technology-by-technology evidence, critical appraisal, implementation prerequisites, and ARA-specific synthesis that support the suggestions made here.

**Table 3:** A deployable AI toolkit for the 2026 IVF clinic.

Technology	Product(s)	Regulatory status	Evidence level	ARA relevance	Tier
T1 Outcome Prediction	Univfy PreIVF Report (US/UK/EU)	Decision support; no SaMD clearance required	Level 2 (22)	Highest: corrects miscalibration; anchors autologous/donor decision	Tier 1 — All clinics
T2 Stimulation Dosing Optimization	CHLOE Stim module (Fairtility, EU); center-specific MLCS	CE marked EU; no FDA clearance	Level 3 (23, 32)	Highest benefit in DOR/ARA: non-linear dose-response best captured by ML	Tier 1 — All clinics
T3 RFID/Electronic Witnessing	RI Witness (CooperSurgical); Vitrolife eWitness; Gidget (Hamilton Thorne)	Not a SaMD; HFEA 2016 compliant; 18-yr safety data	Level 2 (18-yr multicenter observational)	Universal; amplified importance per irreplaceable ARA embryo	Tier 1 — All clinics
T4 AI Ultrasound Monitoring	CycleClarity (select markets); FOLLISCAN (MIM Fertility, EU/US)	Two FDA-cleared platforms; FOLLISCAN CE Class IIa with peer-reviewed multicenter validation	Level 3 (FOLLISCAN multicenter; CycleClarity company data)	No ARA-specific data; visit-reduction benefit applies universally	Tier 1 — Software licensing; FDA-cleared
T6 AI Embryo Assessment (adjunctive)	CHLOE Blast (Fairtility; FDA 510(k)); iDAScore v2 (Vitrolife; CE); KIDScore D5 v3 (Vitrolife; CE)	CHLOE Blast: FDA 510(k) + CE; iDAScore/KIDScore: CE only	Level 1 — divergent (27 [peer-reviewed] failed NI; LOTUS [28] conference report met NI, pending peer-review)	AUC 0.596 → 0.768 with age (1); highest discriminatory gain at ≥40	Tier 2 — Time-lapse incubator required
T7 Endometrial Receptivity AI (pilot)	Research-stage platforms (e.g., EndoClassify model; Bornaun composite framework)	No clearance (FDA or CE-MDR) for endometrial AI	Level 3 ((30); single-center retrospective)	Relevant for RIF; ARA benefit hypothesized but not validated	Tier 3 — Research capacity required

Six AI tools stratified by deployment tier: Tier 1 = software licensing only (no capital hardware; includes FDA-cleared T4 CycleClarity); Tier 2 = time-lapse incubation infrastructure required; Tier 3 = research capacity required. Tier (infrastructure burden) and Wave (timeline to widespread adoption) are independent axes: T4 has FDA-cleared platforms (Tier 1 infrastructure) but its evidence maturity and validation status place broad deployment in Wave 2 (5–10 years). Columns: product name(s), regulatory status, evidence level (OCEBM), and ARA relevance. All AI tools should be implemented as adjuncts to, not replacements for, clinical judgment.

### Tier 1: entry toolkit (software-only, no hardware investment)

The Tier 1 toolkit requires only software licensing, electronic health record (EHR) integration, and staff training. First, RFID-based electronic witnessing (T3) systems (e.g., RI Witness (CooperSurgical), Vitrolife eWitness, and Gidget (Hamilton Thorne)) are all commercially deployed (33). For example, RI Witness uses passive RFID tags and workstation readers to provide continuous automatic monitoring, and the per-cycle licensing makes it accessible at any volume (33). Second, AI outcome prediction (T1) systems such as the Univfy PreIVF ML models has been validated in a 2025 study demonstrating significantly improved discriminatory performance over national registry age-based prediction (22, 34). This model could inform counseling on expectations, multi-cycle planning, and the autologous/donor decision. Third, AI stimulation dosing (T2) system is available as a module within the CHLOE platform (Fairtility) or built as a center-specific starting-dose ML model from existing electronic medical record (EMR) data (23, 32).

### Tier 2: mid-level toolkit (time-lapse equipped)

Clinics with time-lapse infrastructure can immediately add the AI embryo assessment tool (T6). CHLOE Blast (Fairtility; FDA 510(k) cleared September 2025; CE marked; building on the algorithm originally described by 35) is the first FDA-cleared ML clinical decision support tool for embryo assessment. Vitrolife offers iDAScore v2 (36) and KIDScore D5 v3 (both CE marked), however, the 14-center iDAScore RCT failed to demonstrate non-inferiority for clinical pregnancy rate (risk difference −1.7%, 95% CI −7.7 to 4.3; 27). These tools should remain adjuncts to embryologist assessment until head-to-head live birth endpoint data are available (see the Evidence Quality section for critical appraisal of divergent RCT evidence).

### Tier 3: advanced toolkit (research-capable)

High-volume centers (>1,000 cycles/year) with established data infrastructure and research programs can extend the toolkit to include AI-assisted endometrial receptivity assessment (T7). While no AI endometrial tool carries FDA or CE-mark regulatory clearance specifically for endometrial receptivity assessment as of April 2026, validated AI models integrating endometrial pattern recognition with embryo quality scoring have been demonstrated in published research and are entering the translational pipeline (37). These centers can also participate in multicenter validation trials for T5b (AI sperm selection, exemplified by the Carrión-Sisternas et al. (26) platform), contributing to the evidence base. Tier 3 clinics should additionally invest in the EHR and laboratory information management system (LIMS) infrastructure to enable multi-modal AI fusion (3, 38). The infrastructure investment required at this tier will require one-time setup cost plus ongoing annual maintenance.

### The ARA-specific toolkit: a clinical cascade in 2026

For IVF physicians managing predominantly ARA patients (≥40 years), the deployment cascade in 2026 could combine Tier 1 tools with T6 adjunctive embryo assessment: T1 prognosis counseling at the first visit, T2 stimulation dosing optimization, T6 embryo prioritization in time-lapse-equipped centers, and universal T3 electronic witnessing (with amplified medicolegal importance in ARA cases where each embryo is irreplaceable). The ARA Patient: Integrated Clinical Synthesis chapter walks this cascade through a representative composite ARA case (Mrs. K) and presents the underlying ARA-AI amplification mechanism.

### Three questions for AI procurement

Before committing to any AI integration contract, clinics should address three foundational questions concerning data ownership and portability, algorithmic drift monitoring obligations, and uptime service-level agreements with manual fallback protocols. These questions, and their analytical rationale, are detailed in the Procurement section.

## The nine AI technologies by deployment readiness

**Figure 1:**
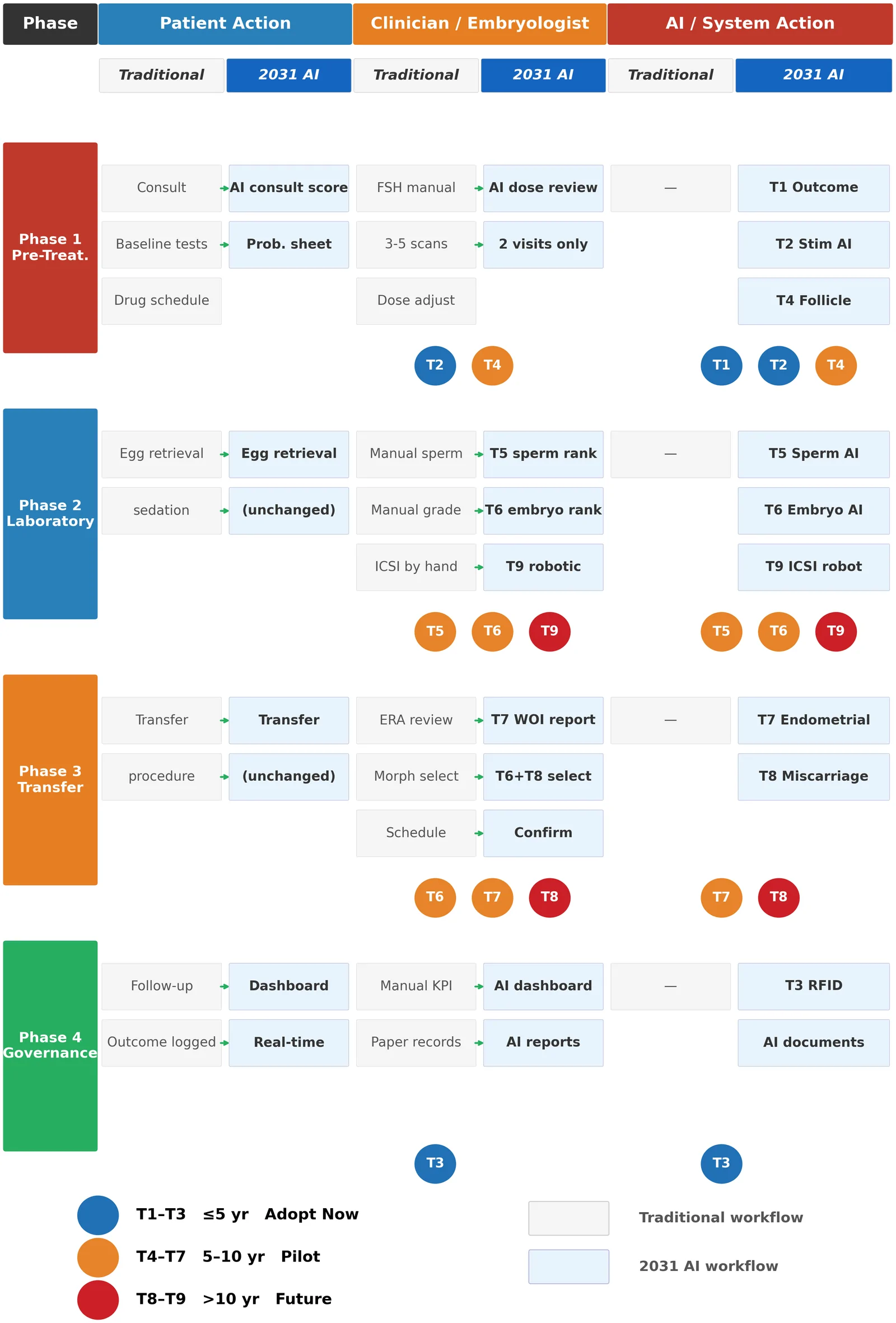
Swimlane comparison of traditional IVF protocol versus 2031 AI-integrated protocol. Three actor lanes (Patient Action; Clinician/Embryologist; AI/System Action) across four clinical phases (Pre-Treatment, Laboratory, Transfer, Governance). Left sub-cell = traditional workflow (light grey); right sub-cell = projected 2031 AI workflow (light blue). Green arrows indicate workflow transitions from traditional to AI-augmented steps. Circular T-number badges indicate the AI technology relevant to each step; badge color encodes the three deployment waves: blue = T1–T3 (Adopt Now, ≤5 yr); amber = T4–T7 (Pilot, 5–10 yr); red = T8–T9 (Future, >10 yr). T3 RFID electronic witnessing is active across all four phases as a laboratory quality-management layer and is shown as blue T3 badges under Phase 4 (Governance).

The nine technologies are organized into three deployment waves reflecting their readiness for clinical adoption. Wave 1 technologies (≤5 years) are software-only tools deployable within 12–18 months in adequately resourced centers; widespread adoption nevertheless depends on payer-reimbursement decisions, embryologist and clinician training pathways, and electronic medical record integration—non-technology factors that can delay clinical translation independently of evidence maturity. Wave 2 technologies (5–10 years) require hardware augmentation and multicenter validation. Wave 3 technologies (>10 years) face fundamental engineering or ethical barriers. As a secondary classification, these technologies are also assigned to different phases within the standard IVF protocol and are summarized in **Supplementary Table 3**. Across the IVF clinical protocol, the nine technologies apply at different stages: T1 anchors pre-cycle counseling and prognostic decision-making; T2 supports the ovarian stimulation phase; T4 supports the follicular monitoring phase; T3 spans all laboratory steps as a quality-tracking layer; T5, T6, T8, and T9 act in the laboratory and embryo selection phase; and T7 supports the transfer-preparation phase through endometrial receptivity assessment (**Supplementary Table 3**). In addition, the traditional versus AI-integrated workflows are illustrated in **Figure 1** (swimlane diagram), with full clinical-stage mappings detailed in **Supplementary Table 5**. The remainder of this chapter applies the deployment-wave organization.

### Wave 1 (≤5 years): deployment-ready technologies

#### T1: AI outcome prediction and patient counseling

##### T1 | deployment timeline: ≤5 years | OCEBM: level 2 | NASSS: simple–complicated | infrastructure: software-only

Holistic outcome prediction—integrating clinical, demographic, and treatment variables to predict live birth probability—is among the most immediately deployable AI applications in IVF: no new hardware is required, and existing EHR data suffice. Yao et al. (22) validated this approach using first-IVF cycle data from 4,635 patients across six fertility centers. Machine learning center-specific (MLCS) models significantly improved discrimination over the SART registry model at the 50% live birth probability threshold (p<0.05), reducing both false positives and false negatives and more appropriately assigning patients to higher LBP tiers.

Sadegh-Zadeh et al. (39) compared multiple ML paradigms, with ensemble methods achieving 96.35% accuracy (Logit Boost). Complementarily, an optimization pipeline combining particle swarm optimization (PSO) with a transformer architecture for tabular data achieved 97% accuracy and AUC 0.98 on UK HFEA registry data (40). These atypically high metrics raise overfitting concerns; independent external validation in a multicenter prospective cohort is essential before clinical application.

A PROSPERO-registered systematic review identified 116 clinical decision-support algorithms (CDSAs) across reproductive medicine, of which 11 had immediate clinical-management or laboratory-practice value (34). By 2031, center-specific prognostic models could become routine counseling components in well-resourced clinics, contingent on prospective studies showing AI-guided counseling improves patient decision quality (see Priority 5).

#### T2: AI stimulation dosing optimization

##### T2 | deployment timeline: ≤5 years | OCEBM: level 3 | NASSS: simple–complicated | infrastructure: software-only

Follicle-stimulating hormone (FSH) dosing remains highly operator-dependent (23, 32). ML dose optimization falls into two categories: (a) starting-dose models using baseline patient characteristics (age, BMI, AMH, AFC) to recommend initial FSH doses (23, 32); and (b) dynamic adjustment models incorporating real-time follicle measurements and hormone levels during stimulation (23, 32, 41, 42). Notably, the only completed prospective multicenter RCT protocol for AI-guided stimulation dosing (IDoser; NCT05948293) was withdrawn prior to enrollment (43), leaving T2’s Wave 1 deployment timeline supported by retrospective evidence only.

By 2031, AI-assisted stimulation planning could become routine in clinics with mature EHR integration, with physicians reviewing algorithm recommendations rather than manually calculating daily doses (23, 32). The next evolution of T2 is pharmacogenomic integration: ML models incorporating FSH receptor (FSHR) gene polymorphisms with standard clinical parameters could stratify ovarian sensitivity prior to stimulation onset, enabling genotype-informed starting doses (44). For ARA patients and poor responders, knowing genetic FSH sensitivity before committing to a protocol may substantially reduce costs from inappropriate dosing.

###### Cross-cutting Wave 1 capability: AI-driven clinical documentation

An underappreciated application is automation of documentation-intensive administrative and clinical reporting. AI-generated embryo development reports across a fertility network demonstrated high patient satisfaction and substantial embryologist time savings (10). AI incorporates time-lapse imaging data, morphokinetic annotations, and clinical context into personalized patient-facing reports, replacing hours of manual drafting per cycle. AI platforms can similarly integrate laboratory outcomes, cycle metrics, and QC logs into real-time dashboards (45, 46).

By 2031, AI-automated documentation could become routine in well-resourced centers, provided regulatory classification of AI-generated clinical reports is clarified. Integration with LIMS and EMR remains the primary implementation challenge, requiring middleware development (38). Whether such reports are classified as medical device software or administrative tools varies jurisdictionally and requires clarification.

Personalized embryo development reports and AI-monitored emotional load during treatment represent additional emerging applications (10). By 2031, patients are expected to access real-time treatment progress through personalized platforms, reducing treatment anxiety.

An emerging extension is generative AI for patient communication—systems processing natural-language clinical queries and producing structured analytical outputs. In a blinded comparative study, large language model (LLM) responses to frequently asked fertility questions were rated equivalent in quality and empathy to those of attending gynecologists and embryologists (47, 48). A comparative evaluation of ChatGPT, DeepSeek, and Gemini on 1,473 autologous IVF/ICSI cycles demonstrated above-chance outcome forecasting (49). However, governance frameworks requiring clinician-in-the-loop oversight are essential prerequisites for responsible deployment.

#### T3: RFID sample tracking and laboratory quality management

##### T3 | deployment timeline: ≤5 years | OCEBM: level 2 | NASSS: simple | infrastructure: hardware (mature)

RFID and barcode sample tracking is mature digital infrastructure—not AI per se, but the data backbone for AI-driven laboratory quality analytics—and ensures near-zero identification error in IVF laboratories (33). International AI fertility conference programs have dedicated sessions to RFID-based workflow intelligence (50).

### Wave 2 (5–10 years): technologies awaiting clinical validation

#### T4: AI ultrasound monitoring

##### T4 | deployment timeline: 5–10 years | OCEBM: level 3 | NASSS: complicated | infrastructure: software-only

Follicle monitoring is among the most repetitive IVF tasks; AI-automated systems count, measure, and track follicles across serial scans. CycleClarity (FDA-cleared) demonstrated in 177 IVF cycles that its algorithm outperforms physician subjective judgment for predicting mature oocytes, with scan time reduced 66% on company-reported data; independent peer-reviewed validation is pending (24).

A multicenter validation of the FOLLISCAN (MIM Fertility) AI platform across IVF centers in Poland, Argentina, Colombia, and the US (5,508 transvaginal ultrasound scans, 1,689 patients) demonstrated 98.2% precision and 93.3% F1 score for follicles ≥10 mm, with annotation time reduced 2.5-fold and stable performance across ultrasound systems (25). Prospective validation reported significantly reduced scan duration alongside high agreement with expert sonographer measurements (51).

However, current AI ultrasound applications operate predominantly on two-dimensional imaging with basic numerical extraction (follicle count and size); deeper feature extraction from raw images and integration of three-dimensional ultrasound remain underexplored (3). Most published models also lack explainable AI (XAI) layers, limiting clinical interpretability (3).

By 2031, AI follicle monitoring could become routine wherever ultrasound platforms support integration, with sonographers verifying automated counts rather than measuring each follicle. The 5–10 year placement reflects not weak performance—FOLLISCAN’s multicenter precision and the prospective agreement data are among the strongest measurement-level evidence in Wave 2 (25, 51)—but the distance between measurement accuracy and clinical benefit: the endpoints validated to date are precision, F1, and sonographer agreement, while the only dataset linking AI measurement to mature oocyte yield remains company-reported with independent validation pending (24).

#### T5: AI sperm analysis, selection, and recovery

##### T5 | deployment timeline: 5–10 years | OCEBM: level 3–4 (by subsection) | NASSS: complicated | infrastructure: software/hardware

Three distinct clinical problems are frequently grouped under the single heading of AI for sperm. They differ in aim, in the data and hardware they require, in the endpoint against which they must be validated, and in the maturity of the supporting evidence. Automated semen analysis is a diagnostic task (T5a); AI-assisted selection of individual spermatozoa for injection is a therapeutic selection task (T5b); and AI-guided identification of viable gametes in non-obstructive azoospermia is a retrieval task (T5c). Robotic ICSI (T9) addresses a fourth and separate problem—the mechanics of gamete manipulation and delivery—and is evaluated in Wave 3. These sperm-related domains are graded separately below and viewed side by side in **Table 4**.

**Table 4:** Four sperm-related AI domains compared.

Domain	Clinical aim	Primary input	Hardware requirement	Validation endpoint	Evidence (OCEBM)
T5a AI semen analysis	Diagnostic — characterize the sample	Brightfield or phase-contrast video of prepared semen	Software only; standard microscope and camera	Agreement with reference method (manual/CASA); accuracy, F1, AUC	Level 3
T5b AI sperm selection for ICSI	Therapeutic selection — rank individual spermatozoa for injection	High-magnification real-time video of motile spermatozoa	Software overlay on the existing ICSI micromanipulation rig	Fertilization rate, blastocyst formation, euploidy; live birth not yet reported in an RCT	Level 3
T5c AI-guided sperm recovery (NOA)	Retrieval — find any viable gamete in a sparse sample	High-speed imaging of processed testicular sample	Microfluidic chip plus high-speed imaging platform	Viable sperm recovered per case; detection precision	Level 4
T9 Robotic ICSI	Manipulation and delivery — perform the injection	Oocyte and pipette tracking; positional and force feedback	Dedicated robotic micromanipulator (capital equipment)	Fertilization rate, usable blastocyst rate, live birth; procedure time, oocyte deformation	Level 4

The three subdomains of T5 — semen analysis (T5a), sperm selection for ICSI (T5b), and sperm recovery in non-obstructive azoospermia (NOA)(T5c) — together with robotic ICSI (T9) differ in clinical aim, input data, hardware requirement, and the endpoint against which each must be validated. Evidence levels follow the OCEBM-adapted hierarchy defined in the Evidence Quality section (Level 3 = retrospective cohort; Level 4 = case series or proof-of-concept). T5a and T5c are validated predominantly against surrogate endpoints — reference-method agreement, classification accuracy, or detection precision — rather than against clinical outcomes. CASA, computer-assisted sperm analysis.

###### T5a: AI semen analysis

T5a | aim: diagnostic characterization | OCEBM: level 3 | endpoint: agreement with reference method; classification metrics

Automated semen analysis addresses the diagnostic question of what a sample contains: concentration, total and progressive motility, kinematic parameters, and morphology. While computer-assisted sperm analysis has long suffered from inter-laboratory variability, early work has established the feasibility of AI-based sperm and semen analysis (52–54). Convolutional and ensemble architectures now classify head morphology from both stained and live unstained preparations (55–57). Integrated computer-vision systems report concentration, motility, and kinematics from a single acquisition (58), and trajectory-based models refine motility classification specifically (59). Contemporary reviews of the andrology-AI field document rapid methodological progress alongside persistent gaps in external validation (60–62). Critically, this literature is validated against agreement with a reference method and against classification metrics—accuracy, F1, AUC—rather than against clinical outcomes, which is why T5a informs diagnosis and triage but does not by itself alter treatment outcome.

###### T5b: AI sperm selection for ICSI

T5b | aim: therapeutic selection | OCEBM: level 3 | endpoint: fertilization, blastocyst formation, live birth

Sperm selection during ICSI conventionally relies on embryologist subjective judgment, introducing inter-operator variability. Building on early AI-based sperm/semen analysis approaches (52–54), recent deep learning models analyze sperm morphology and motility to provide objective scoring (9, 63). A single-center observational study (n=102 couples, 628 oocytes) showed a non-significant trend toward higher blastocyst formation rate (72.14% vs 67.41%) and euploid blastocyst rate (57.14% vs 49.06%) with AI versus manual selection (26). A prospective sibling-oocyte comparison of automated single-sperm selection software reported fertilization and early developmental outcomes but did not include a live-birth endpoint (64), and object-detection approaches have been applied to support real-time selection (65). A systematic review of AI for sperm selection concluded that no platform has yet demonstrated improved clinical outcomes in randomized comparison (66, 67). No AI sperm selection study has yet reported live birth as a prospective primary endpoint in an RCT.

By 2031, AI-assisted sperm selection could become an adjunct at the ICSI workstation, particularly in male factor infertility and compromised sperm quality. Full clinical deployment of integrated morphokinetic sperm AI remains constrained by regulatory classification uncertainty and the absence of RCT evidence with live birth endpoints, placing T5b at the outer boundary of the 5–10 year horizon rather than among earliest Wave 2 technologies.

###### T5c: AI-guided sperm recovery in non-obstructive azoospermia

T5c | aim: gamete retrieval | OCEBM: level 4 | endpoint: viable sperm recovered per case; detection precision

The STAR (Sperm Tracking and Recovery) system addresses non-obstructive azoospermia (NOA)—a population for whom conventional AI sperm-selection tools provide no value (68)(**Table 4**). It integrates a microfluidic chip, a high-speed imaging platform, and a detection model triggering microfluidic gating into a recovery volume. The system achieved precision 0.89, and its first clinical application yielded a single confirmed pregnancy in a man with non-obstructive azoospermia—a proof-of-principle result demonstrating technical feasibility but not interpretable as evidence of clinical efficacy without prospective case series data (68). Distinct from AI that characterizes or ranks spermatozoa already present (T5a, T5b) and from robotic ICSI (T9), STAR targets the sperm-finding step and warrants inclusion in the 5–10-year translational agenda for severe male factor infertility.

#### T6: AI embryo assessment and selection

##### T6 | deployment timeline: 5–10 years | OCEBM: level 1 | NASSS: complicated | infrastructure: software-only

AI embryo assessment encompasses time-lapse morphokinetic systems and static image deep learning models (26, 69–71). One platform integrating Day 1 and Day 3 embryo images with clinical metadata across four prediction modules demonstrated feasibility of a unified system covering the full embryo assessment lifecycle (71). AI models on pre-vitrification oocyte images are also being developed to predict thaw survival and blastocyst conversion (72).

Beyond morphokinetic scoring, AI-integrated metabolic imaging is emerging as a distinct assessment modality. METAPHOR (Metabolic Evaluation through Phasor-based Hyperspectral Imaging and Organelle Recognition) uses two-photon infrared illumination of endogenous metabolic fluorophores with AI phasor analysis to generate label-free metabolic fingerprints, achieving AUC 0.962 for oocyte age discrimination and 0.822 for blastocyst formation prediction in mouse models (73). Preliminary ESHRE 2024 human data demonstrated inner cell mass versus trophectoderm metabolic heterogeneity invisible to brightfield microscopy (74). Micro-magnetic resonance spectroscopy achieved non-destructive single-cell metabolic fingerprinting in mammalian embryos (75), and hyperspectral microscopy with ML identified distinct signatures in aneuploid versus euploid embryos (76). Large-scale safety studies remain prerequisite before adoption.

Importantly, the clinical utility of these AI systems is not uniformly distributed; their discriminatory advantage is profoundly amplified in ARA patients. This reflects a biological reality: aneuploidy rates rise from ~20–30% at age 32 to >55% at age 40 and 80–90% by age 43–45 (2). In younger patients, a high proportion of euploid embryos implant regardless of subtle AI-detected features; in ARA patients, the wider embryo-to-embryo quality variation amplifies the AI system’s discriminatory advantage (77).

This age-stratified pattern, however, derives from a single retrospective analysis of one AI platform (KIDScore D5; 1) in one Japanese cohort, has not been independently replicated on other platforms, and was not observed in the iDAScore RCT (27), which failed non-inferiority across all age groups. Prospective age-stratified multicenter validation across multiple AI platforms is therefore a prerequisite before ARA patients can be designated as the primary beneficiary population. Throughout this review, the ARA-amplification thesis is treated as a hypothesis-generating observation rather than an established clinical principle, and the deployment-readiness analysis stands independently of its eventual confirmation.

By 2031, embryo assessment is expected to integrate multiple data modalities through AI fusion algorithms, contingent on large-scale RCT validation with live birth endpoints (3). CHLOE Blast and EmbryoScope-integrated platforms (KIDScore, iDAScore) are available for adjunctive deployment in time-lapse-equipped centers (36, 78), with evidential and clinical limitations described in the AI Toolkit section.

#### T7: AI endometrial receptivity assessment

##### T7 | deployment timeline: 5–10 years | OCEBM: level 3 | NASSS: complicated | infrastructure: software-only

The Window of Implantation (WOI), typically days 19–21, shows individual variation that can cause implantation failure undetected by standard morphological assessment. AI-based endometrial assessment combining multi-modal ultrasound segmentation with embryo quality classification outperforms conventional assessment for live birth (composite AUC 0.94 in single-center retrospective data with synthetic augmentation—rated High RoB in **Table 2**) (30, 79). By 2031, AI endometrial assessment is expected to complement embryo scoring in an implantation prediction framework for recurrent implantation failure (RIF) patients, potentially reducing frozen embryo transfer (FET) failure-associated costs (80). T7 deployment requires only software overlays on existing ultrasound platforms, but prospective multicenter trials with live birth endpoints are essential for mainstream adoption.

### Wave 3 (>10 years): technologies beyond current capability

#### T8: AI miscarriage risk prediction

##### T8 | deployment timeline: >10 years | OCEBM: level 3 | NASSS: complex | infrastructure: software-only

First-trimester miscarriage rates are strongly age-dependent: approximately 10–15% at age <35, rising to >40% in ARA patients (≥40 years), driven predominantly by chromosomal aneuploidy (81). In a multicenter retrospective study of 391 ICSI patients, an ML classifier developed for first-trimester miscarriage prediction from time-lapse images of preimplantation embryos used a minimal subset of six non-redundant morphodynamic features (31). Miscarriage could be predicted by day 3 post-ICSI fertilization with AUC ~0.7, even though all embryos had been selected by experienced embryologists using standard quality markers (31). These data suggest miscarriage-related features are detectable at the earliest preimplantation stages but invisible to conventional morphological assessment.

The ERICA algorithm (82) categorized 172 blastocysts by quality group (optimal/good/fair/poor) and correlated the ranking with presence or absence of fetal heartbeat versus spontaneous abortion, yielding a classification accuracy of 67.4%. A 2025 dual-center machine-learning analysis of 1,664 single vitrified-warmed blastocyst transfers (308 early miscarriage cases) achieved AUC 0.836 (Voting Classifier) and 0.831 (Gradient Boosting) for early miscarriage prediction, substantially outperforming traditional logistic regression (AUC 0.584) (83).

By 2031, miscarriage risk prediction is expected to complement implantation scoring as a dual-assessment framework, with each avoided miscarriage reducing management costs and patient suffering (80). However, the modest AUC (~0.7) of preimplantation models requires substantially larger prospective cohorts before deployment. The ethical framework for withholding transfer based on preimplantation risk scores must also develop in parallel with technical validation.

#### T9: robotic ICSI

##### T9 | deployment timeline: >10 years | OCEBM: level 4 | NASSS: complex | infrastructure: hardware-dependent

T9 encompasses two technically distinct paradigms: (i) automated ICSI, in which a robotic device performs injection at the same physical site as the embryologist team, and (ii) remote (teleoperated) ICSI, in which the operator is physically separated from the procedure. These differ in regulatory pathway, infrastructure requirements, and clinical-operational implications, and are reported here as separate milestones. In 2025, automated-ICSI proof-of-concept demonstrated that three integrated robotic systems achieved 64.3% fertilization, 42.2% usable blastocyst rate, and 5 live births from 12 transfers (11, 12). In the same year, the world’s first remote-ICSI live birth involved operators separated by 3,700 km (9). Overture Life signed a July 2025 commercial deployment agreement with Memorial Hospital Istanbul targeting >6,000 cycles annually (84). Parallel automation efforts include automated oocyte vitrification devices (85).

## Engineering constraints on routine deployment

Beyond throughput, three engineering constraints separate a demonstrated live birth from a routine clinical service, and none is yet resolved. First, manipulation accuracy: ICSI requires the injection pipette to be positioned and driven through the oolemma within tolerances measured in micrometers, with penetration depth and needle trajectory controlled against an oocyte that is itself deformable and free to rotate. An automated system must therefore recover the pose of both pipette and oocyte in real time and must do so reliably enough that failure is detectable before damage occurs. Second, motion and path planning: the trajectory taken by the micropipette is not merely a matter of reaching the target, because the surrounding cytoplasm behaves viscoelastically and the path itself determines the mechanical load imposed. Path-planning methods that explicitly optimize the trajectory to minimize both path length and induced deformation have been formulated for viscoelastic micro-biological particles (86), but have not been validated in human oocytes. Third, cell deformation and gamete safety: deformation follows directly from the applied trajectory and force, and the tolerance of the human oocyte to prolonged mechanical loading during automated handling has not been characterized. Real-time control approaches developed toward ICSI automation illustrate both the feasibility of closed-loop manipulation and the difficulty of guaranteeing safety margins (87).

However, critical limitations remain: automated ICSI averages 8.7 min/oocyte versus ~2 min manual, and post-denudation still requires embryologist intervention. Prolonged N-2-hydroxyethylpiperazine-N-2-ethanesulfonic acid (HEPES)-buffer exposure during manipulation may also adversely affect embryo development (12, 88). By 2031, robotic ICSI is expected in select high-volume centers, but full adoption requires further engineering iteration. The >10-year placement of T9 therefore follows from the conjunction of Level 4 evidence—proof-of-concept series without comparative trial—and these unresolved engineering constraints, rather than from the evidence grade alone.

## The patient perspective

The technology evidence above establishes what AI can do; the patient perspective establishes what is acceptable to those receiving it. Cromack et al. (89) surveyed 200 fertility patients on AI perceptions in infertility care. While 55% generally supported AI in medicine, only 46% trusted AI-informed reproductive care—a meaningful trust gap. Patients consistently want AI to enhance, not supplant, the physician-patient relationship, and value transparency about when and how AI contributes to treatment decisions.

Beyond clinical acceptability, the same survey found concerns extending to algorithmic opacity: patients want to know why an AI system ranked one embryo above another and whether it accounts for individual history rather than population averages. Equity is an additional dimension—patients at resource-limited clinics without time-lapse infrastructure or LIMS integration may be unable to access AI-augmented care. Furthermore, algorithmic matching and facial recognition raise profound questions about autonomy, identity, and informed consent (69). For the 2031 clinic, patient-facing AI applications will require careful consent frameworks explaining AI’s role, and a key unresolved question is whether patients should be able to opt out of AI-assisted embryo selection without penalty or pressure. Collectively, these governance gaps represent organizational prerequisites that must be resolved before the 2031 AI-integrated clinic can function as trustworthy routine practice.

## Evidence quality and critical appraisal

Before these organizational prerequisites can be addressed, the evidence base itself requires critical scrutiny. This chapter provides a critical appraisal of the nine technologies along four complementary dimensions. The Evidence Quality Assessment subsection evaluates the strength of the underlying clinical evidence using an OCEBM-adapted hierarchy and a study-by-study quality assessment. The Research Priorities subsection translates the resulting evidence gaps into five concrete research priorities for the 2026–2031 agenda. The Deployment-Timeline Confidence subsection evaluates how confident the field can be in the projected deployment timelines. The Resource Impact Analysis subsection closes with a resource-impact analysis covering time, cost, training burden, and clinical-quality dimensions. Together they form the bridge between the technology evidence base and the implementation prerequisites that follow.

### Quality assessment of key clinical studies

Each technology was classified by an OCEBM-adapted hierarchy (90): Level 1 = multicenter RCT; Level 2 = single-center RCT or multicenter observational; Level 3 = retrospective cohort; Level 4 = case series or proof-of-concept; Level 5 = expert opinion or conference report only. Only AI embryo selection has achieved Level 1 evidence; most technologies remain at Level 2–3, while the sperm-related and robotic domains (T5b, T5c, T9) sit at Level 3–4 and rest predominantly on surrogate endpoints—classification accuracy, F1 score, or agreement with a reference method—rather than on clinical outcomes such as live birth. Within this hierarchy, **Table 2** presents a quality assessment of the key clinical studies across five dimensions: study design, sample size, primary endpoint, key result, and risk of bias (RoB; encoded using Cochrane-style traffic light shading: + green = Low,? yellow = Moderate, − red = High). **Supplementary Table 2** lists the full evidence-grading assignments.

#### Key observation

The two largest RCTs on AI embryo selection produced divergent results. The Illingworth et al. (27) trial used a time-lapse deep learning algorithm (iDAScore) across 14 clinics and found clinical pregnancy rate (CPR) of 46.5% versus 48.2% (risk difference −1.7%, 95% CI −7.7 to 4.3), failing non-inferiority. The LOTUS (Live birth Outcomes from Time-lapse Using AI) trial (28, building on the AI-based viability prediction model originally developed by 91) used a static image-based AI system across 7 US clinics and found CPR of 72.9% versus 68.0%, meeting non-inferiority. The divergence likely reflects differences in AI architecture (video-based vs. static image), time-lapse hardware dependency, control group standardization, and patient population. This underscores that AI embryo selection is platform-dependent and cannot be treated as a uniform technology category. These platform-specific findings translate into clear clinical guidance: practitioners should evaluate specific AI embryo selection platforms against their patient demographics (ARA cohort vs general population), endpoint requirements (live birth vs implantation), and evidence depth (peer-reviewed vs conference-report) rather than treating ‘AI embryo selection’ as a uniform clinical category.

Additional quality notes include: (1) Endometrial receptivity AI: the composite AUC 0.94 (30) carries high RoB for the reasons given in the T7 section; explainable-AI layers (e.g., saliency/attribution maps) are an additional prerequisite for deployment. (2) Miscarriage prediction: as documented in the T8 Miscarriage Prediction section, preimplantation AUC ~0.7 indicates modest discrimination; post-transfer ultrasound-based models achieve higher accuracy (AUC 0.97) but operate at a later timepoint (92). (3) Outcome prediction: the MLCS models (22) are retrospectively validated across 6 centers but have not yet been prospectively tested for their impact on patient decision-making or clinical outcomes; temporal data drift is a recognized concern.

### Research priorities for the 2026–2031 agenda

At least five research priorities have emerged from the tensions between current evidence and forward-looking projections of the 2031 IVF clinic. Each tension is paired with a concrete research action that, if undertaken systematically by ESHRE, ASRM, and national fertility societies, would resolve the tension and strengthen the evidence base supporting clinical adoption of these technologies. Priority 1 is a technology for which standards exist and require implementation; Priorities 2–5 are open scientific questions requiring new evidence generation.

#### Priority 1: platform-specific live birth endpoint validation for AI embryo selection

Our projection assumes AI embryo assessment will be standard by 2031. Current RCT evidence supports non-inferiority — not superiority — for one platform (28) and fails to support it for another (27). Non-randomized observational data from the single-center icONE pilot suggesting CPR of 77.3% (93, conference abstract reporting preliminary results) cannot be extrapolated to RCT settings and should not inform projections. **Research action:** The projection is re-framed as “non-inferior with workflow efficiency benefits, pending platform-specific live birth endpoint validation” rather than “superior outcomes”. Clinics cannot extrapolate efficacy data across platforms.

### Priorities 2–5 — open scientific questions for new evidence generation

#### Priority 2: health-economic modeling and cost-effectiveness analyses

Cost is the top adoption barrier (38.01% of respondents, 2025) (10). While no peer-reviewed per-tool cost analysis exists as of April 2026, full-pipeline integration implies cumulative costs across multiple tools that have not been systematically modeled. **Research action:** Because cost-effectiveness data remain insufficient, formal incremental cost-effectiveness ratio (ICER) analysis per technology is a prerequisite for broad adoption (the Implementation Prerequisites section).

#### Priority 3: explainable AI development and standardized training curricula

33.92% of professionals cite lack of training as a barrier (10), and the black-box nature of deep learning models limits trust (3). **Research action:** explainable AI (XAI) development and training curriculum reform are identified as prerequisites.

#### Priority 4: prospective multicenter trials for AI endometrial receptivity assessment

As established in the T7 section, the AUC 0.94 evidence base is single-center and retrospective. The RNA-seq WOI prediction (94) requires invasive biopsy in a prior cycle, adding cost and time not justified outside the RIF population. **Research action:** Prospective multicenter validation with live birth endpoints is the field’s most urgent T7 research priority; until that evidence exists, T7 should be deployed only within supervised research protocols.

#### Priority 5: cross-center generalizability testing for outcome prediction models

The MLCS models (22) demonstrate significant inter-center variation in patient characteristics and live birth outcomes — precisely why center-specific models outperform national registry models. However, this means models require center-specific calibration and periodic revalidation as patient demographics and protocols change. Prospective impact studies assessing whether AI-guided counseling actually changes patient behavior and clinical outcomes are needed. **Research action:** Prospective impact studies — randomized evaluations of AI-guided versus standard counseling, with clinical outcomes as endpoints — are needed before T1 can claim to improve patient decision-making, not merely model performance.

### Confidence assessment of deployment timelines

**Figure 2:**
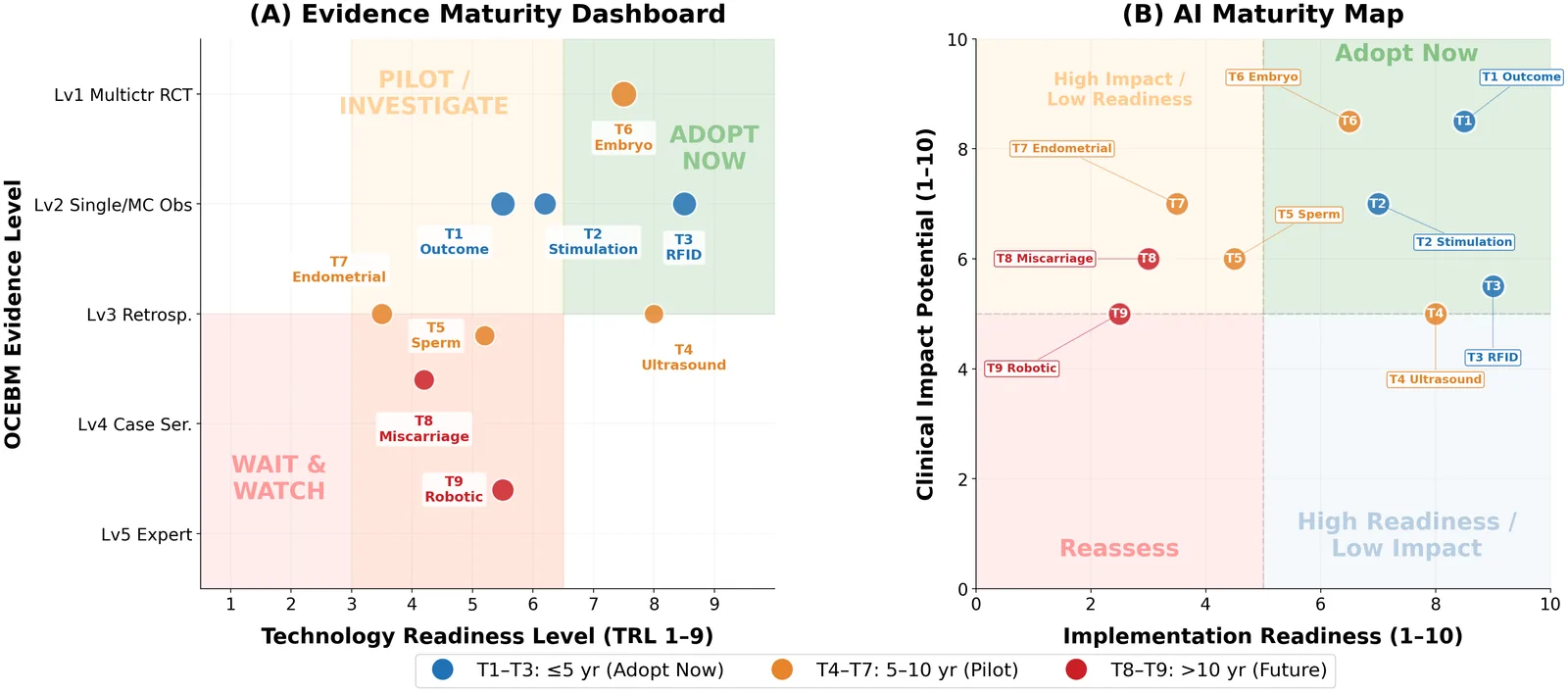
Dual-axis assessment of nine AI technologies (T1–T9). **(A)** Evidence Maturity Dashboard: scatter plot positioning each technology by Technology Readiness Level (TRL 1–9, x-axis) against OCEBM evidence strength (y-axis; Level 1 = multicenter RCT at top, Level 5 = expert opinion at bottom). Three decision zones — Adopt Now (TRL ≥6.5 + OCEBM Level 1–2, green), Pilot/Investigate (moderate TRL or evidence, amber), and Wait & Watch (low TRL + weak evidence, red) — reflect readiness for clinical deployment. Bubble color encodes projected widespread deployment wave, not regulatory clearance status: blue = T1–T3 (≤5 yr, Adopt Now); amber = T4–T7 (5–10 yr, Pilot); red = T8–T9 (>10 yr, Future). T4 (Ultrasound) occupies TRL 7–8 owing to available FDA-cleared platforms but is coded amber because independent multicenter validation and broad deployment are projected at the 5–10 year horizon. T1 (Outcome Prediction) is positioned at TRL 7 consistent with multicenter prospective-validation cohort evidence (Level 2; 22, 39). Exact TRL and OCEBM assignments for all nine technologies are provided in **Supplementary Table 2**. **(B)** AI Maturity Map: quadrant plot positioning each technology by implementation readiness (x-axis, 1–10) against clinical impact potential (y-axis, 1–10). Dashed lines at x = 5 and y = 5 define four quadrants: Adopt Now (high readiness + high impact, upper right), High Impact/Low Readiness (upper left), High Readiness/Low Impact (lower right), and Reassess (lower left).

This section evaluates projected claims against general ML deployment experience. The evidence maturity and TRL positioning of all nine technologies is summarized in **Figure 2** and assigns each technology to a confidence tier.

#### Claims requiring qualification (50–80% confidence)

T4 (AI ultrasound monitoring), T5b (AI sperm selection), and T7 (endometrial AI) carry unresolved generalizability limitations. T4’s 66% scan-time reduction is company-reported without independent replication. T5b evidence derives from a single center with surrogate endpoints. T7’s composite AUC derives from the single retrospective center described in the T7 section.

#### T6 occupies a transitional position

Regulatory-cleared platforms (CHLOE Blast; Embryo Predict (Alife Health, CE marked)) can be deployed adjunctively in well-resourced centers within five years, but full deployment as the primary selection criterion requires the 5–10-year horizon pending resolution of the divergent RCT evidence detailed in the Evidence Quality Assessment subsection. The distinction is between T6 as an available adjunct (≤5 years) and T6 as a definitive replacement for morphology-based selection (5–10 years).

#### Claims assessed as overly optimistic (<50% confidence by 2031)

T8 (miscarriage prediction as a transfer decision tool) and T9 (Robotic ICSI at scale) face barriers that preclude 2031 clinical deployment. T8’s AUC ~0.7 is insufficient for withholding transfer, and the ethical framework for doing so is absent. T9’s 4× speed limitation is a hardware engineering constraint requiring years of iteration beyond current capability.

#### Three-stratum adoption summary

The full technology viability assessment across three adoption strata is summarized in **Supplementary Table 2**. Overall, the first stratum (≤5 years) comprises software-only tools with Level 2–3 OCEBM evidence (T1: Level 2; T2: Level 3; T3: Level 2) and Simple–Complicated NASSS profiles — AI outcome prediction (T1), stimulation dosing (T2), and RFID quality tracking (T3). The second stratum (5–10 years) requires hardware augmentation, multicenter validation, and broader regulatory clearance — ultrasound monitoring (T4), sperm analysis and selection (T5), embryo selection beyond cleared platforms (T6), and endometrial receptivity AI (T7). The third stratum (>10 years), including autonomous miscarriage-based transfer decision support (T8) and robotic ICSI (T9), faces fundamental engineering constraints irrespective of algorithmic progress or unresolved ethical frameworks. The integration feasibility of these technologies heavily depends on three organizational prerequisites — EHR interoperability, LIMS application programming interface (API) access, and SaMD procurement capacity, instead of technology readiness alone. Centers investing in these prerequisites during 2026–2030 can deploy first-stratum tools within 12–18 months.

### Resource impact analysis

The resource impact of each technology is rated across four dimensions: time savings, cost savings, training impact, and clinical quality/standardization (**Supplementary Table 6**). Technologies with the highest immediate resource impact include: (1) AI Outcome Prediction/Counseling (T1) — no hardware required; if AI-guided counseling leads patients to forgo cycles with very low success probability, the theoretical cost avoidance could range from $12,000–$30,000 per avoided cycle in US (80), (2) RFID/Quality Tracking (T3) — mature technology delivering 25% laboratory cost reduction and zero-error sample traceability, (3) AI Endometrial Receptivity Assessment (T7) — avoidance of each failed FET represents $3–6K in direct cost avoidance (80, 95). Future analyses should calculate the Incremental Cost-Effectiveness Ratio (ICER) per technology using Markov decision-tree models (80). On the other hand, in value-based or risk-sharing financing arrangements, time-to-pregnancy technologies (T6, T7) carry substantially higher economic value than in fee-for-service settings. Technologies with negative near-term resource impacts include Robotic systems (denudation, ICSI; T9) that currently increase procedure time and require substantial capital investment. Their resource benefit is realized only at scale with future speed improvements.

## Deployment-ready capabilities and near-term clinical wins

Of the nine AI technologies evaluated, three Tier 1 software-only applications offer immediate clinical implementation value with minimal capital investment. T1 (AI outcome prediction): the Yao et al. (22) multicenter MLCS validation across six US fertility centers (N = 4,635 first IVF cycles) demonstrates PR-AUC 0.75 versus the SART model’s 0.69 (p<0.05), with 23% of patients reclassified to live birth probability ≥50% and 11% to ≥75% — directly translating to higher-fidelity ARA counseling and donor-versus-autologous decision support. T2 (stimulation dosing): Fanton et al. (23) multicenter retrospective evidence (3 US ART centers, N = 18,591 cycles) identifies per-patient dose–response curves that improve MII oocyte yield in low-prognosis patients, with an interpretable starting-dose model that facilitates clinician trust. T3 (RFID quality tracking): established sample chain-of-custody and witnessing technology, deployable in any IVF laboratory within standard quality assurance budgets. Two Tier 2 applications additionally offer immediate adjunctive value where infrastructure permits: T4 (AI ultrasound monitoring) via FOLLISCAN (25; peer-reviewed multicenter validation across N = 1,689 cycles) provides radiation-free, AI-assisted ovarian monitoring with validated precision and F1 metrics; and T6 (AI embryo assessment) for ARA patients, where Kato et al. (1) demonstrates AUC progression from 0.596 (age <35) to 0.768 (age ≥43) — a 29% relative gain in discriminatory power precisely in the population where embryo selection decisions carry the highest clinical stakes.

## AI failure modes and risk mitigation

Even when evidence and economics favor adoption, AI systems introduce failure modes distinct from conventional medical devices and require dedicated mitigation. Unlike obvious equipment failures, AI models fail silently—producing confident but incorrect outputs that clinicians may act upon unaware (31). These failures include:

### Algorithmic drift and distribution shift

Models trained on historical data degrade when patient populations, protocols, or equipment change. An embryo scoring model trained on patients aged 28–38 may underperform for those >40 or with endometriosis. Mitigation: continuous performance monitoring with automated alerts; periodic retraining; reporting of model version and training demographics (3, 31, 38).

### System downtime

Cloud-dependent AI systems are vulnerable to outages. In time-sensitive IVF decisions (trigger timing, transfer scheduling), outages force reversion to manual protocols. Mitigation: documented manual fallback protocols for AI-integrated workflows; on-premise deployment with cloud sync; contractual service-level agreement (SLA) (32, 78).

### Adversarial and edge-case failures

Deep learning models can be sensitive to image artifacts (bubbles, debris, optical aberrations) that do not affect human assessment, and encounter edge cases like arrested development, mosaic embryos, and culture artifacts. Mitigation: adversarial testing during validation; human oversight for edge cases; transparent confidence intervals on all AI outputs (3, 23, 37).

### Accountability gaps

When AI contributes to a harmful clinical decision, responsibility among clinician, institution, and vendor remains legally unresolved. The EU AI Act classifies certain AI medical devices as high-risk, but liability remains ambiguous. Under the learned intermediary doctrine—which holds the treating clinician, not the manufacturer, primarily liable—the hospital currently bears malpractice exposure when AI informs embryo selection or transfer decisions. Until insurers establish explicit underwriting frameworks for AI-augmented ART, institutional risk committees will likely restrict these tools to observational or adjunctive roles (78).

Each of these failure modes intersects with one or more of the implementation prerequisites considered in the next chapter.

## Implementation prerequisites

Beyond the failure modes addressed in the preceding chapter, realization of the 2031 vision could require progress across four prerequisite clusters that span evidence generation, regulatory pathways, ethics, and workforce and data infrastructure. Procurement considerations, addressed briefly in the section that follows, constitute the operational layer that determines whether AI deployment actually delivers clinical value.

### Evidence generation and validation standards

To realize the 2031 vision, at least 5–10 multicenter RCTs covering diverse platforms, populations, and geographies with live birth as primary endpoint are needed (71). The ‘Croatia Consensus’ (a proposed international validation framework; 63 for AI in embryo assessment) validation framework needs to be endorsed by ESHRE and ASRM within five years (30). For miscarriage prediction, the ethical framework for withholding transfer must be developed in parallel with technical validation.

### Regulatory pathways and jurisdictional harmonization

Regulatory classification of AI-based IVF tools varies substantially across the six jurisdictions assessed in **Table 5** (FDA, EU MDR, UK HFEA, Australia TGA, Japan PMDA, China NMPA) (97). A fundamental divergence — between the FDA’s Predetermined Change Control Plan (PCCP), which permits continuously-learning algorithm updates without resubmission, and the EU AI Act, which requires conformity reassessment for material modifications — creates a higher barrier to adaptive AI in Europe (21, 69). Cross-border data-flow compliance and robotic-system safety certification pathways remain underdeveloped across all jurisdictions, and national IVF data-sharing frameworks analogous to SART or HFEA are absent outside the US and UK, limiting population-specific validation.

**Table 5:** Regulatory classification of AI-based IVF tools across six jurisdictions.

Jurisdiction	Regulatory body	AI classification	Clearance pathway	Current status (April, 2026)
United States	FDA (CDRH)	SaMD Class II	510(k) or *De Novo*	CHLOE Blast cleared Sep 2025
European Union	Notified Bodies (EU MDR)	Class IIa medical device	CE marking + EU AI Act compliance	Embryo Predict (96) CE marked Oct 2025
United Kingdom	MHRA + HFEA	SaMD; HFEA oversight	UK Conformity Assessed (UKCA)	No AI embryo tool independently cleared
Australia	Therapeutic Goods Administration	Class IIa (IVD/SaMD)	Conformity assessment	No IVF-specific AI clearance
Japan	Pharmaceuticals and Medical Devices Agency	SaMD program (DASH)	DASH digital health pathway	No IVF-specific AI clearance
China	National Medical Products Administration	Class II/III AI device	Registration certificate	No AI-IVF clearances identified; Class III SaMD pathway applies; PGT-A standard published 2025

Regulatory body, AI classification pathway, clearance status as of April 2026, and key jurisdictional notes for each of the six regulatory frameworks assessed: FDA (United States), EU MDR (European Union), HFEA (United Kingdom), TGA (Australia), PMDA (Japan), and NMPA (China).

### Ethical prerequisites

Clear frameworks are needed for algorithmic transparency, informed consent for AI-assisted embryo selection, and data privacy in AI-integrated workflows in most countries (98, 99). The ethical dimensions of AI in family creation require ongoing public engagement, and professional society position statements would provide the starting points for these issues (70).

### Workforce and data infrastructure

AI competency, including data science literacy, appropriate use of AI tools, and robotic system supervision, must be integrated into reproductive medicine training for clinicians and embryologists (45, 78). Embryologists’ roles will transition from manual operators to quality control supervisors and system optimization experts, and this requires entirely new training curricula. The multi-modal embryo assessment cannot be realized without a system that unifies imaging, EHR, and genetic testing data standards (3, 38).

Because deployment timelines are frequently determined by IT infrastructure (e.g., EHR/LIMS integration, cybersecurity audits, and informatics labor), rather than AI readiness, a lack in this aspect could extend timelines by 1–2 years, representing a hidden cost absent from most implementation cost estimates (78). In addition, manual fallback protocols and continuous AI proficiency assessments must be established to ensure efficient operation (38).

## Operational layer: procurement and integration contracts

Many real-world AI deployments in healthcare systems fail at procurement. Company lock-in, ambiguous data ownership, undefined drift monitoring obligations, and unenforceable performance guarantees translate into operational risk that is invisible in technology readiness assessment. Software-based AI (T1, T2, T4, T5, T6, T7, T8) is typically structured as Software-as-a-Service (SaaS) operating expenditure — though T4 and T5 may additionally require proprietary imaging hardware with associated capital cost. Robotics (T9) could be delivered as a capital expenditure with multi-year depreciation, while RFID systems (T3) have hybrid arrangements. The appropriate contract architecture and total cost of ownership vary substantially by clinic volume, jurisdiction, payer system, and institutional procurement infrastructure. Three questions, however, are common to all institutional contracts. First — data ownership and portability: who owns the clinic’s data, the models trained on it, and what happens if the contract terminates or the company is acquired? Without explicit provisions, clinics commonly find that data fed to a company’s training pipeline cannot be retrieved or repurposed. Second — drift monitoring and update cadence: who is responsible for monitoring algorithmic drift over time, and who validates updates before they reach clinical workflow? (78). Third — uptime, performance, and remediation: what service-level agreement governs uptime, what performance benchmarks must the model maintain, and what compensation is owed for downtime or performance degradation? Beyond these three, procurement decisions are mostly institution-specific and require dedicated finance, legal, and informatics input that lies outside the scope of this review.

## The ARA patient: integrated clinical synthesis

The preceding chapters have evaluated nine AI technologies as discrete entities. This chapter integrates that analysis to address a common clinical question: how does the AI-integrated IVF clinic actually function for the patient population in whom AI delivers the greatest value? This review suggests that patients of advanced reproductive age (≥40 years) represent that particular population. AI amplification for ARA patients could be realized across multiple technologies: discriminatory advantage rises with maternal age in T6 embryo selection, individualized stimulation calibration matters more in diminished ovarian reserve in T2, and prognosis miscalibration carries higher stakes when treatment windows are short in T1. This means that a coherent ARA-specific clinical setting serves both as a stress test for the toolkit and as a practical guide for the IVF physicians for whom these patients represent a substantial fraction of their caseload.

**Figure 3:**
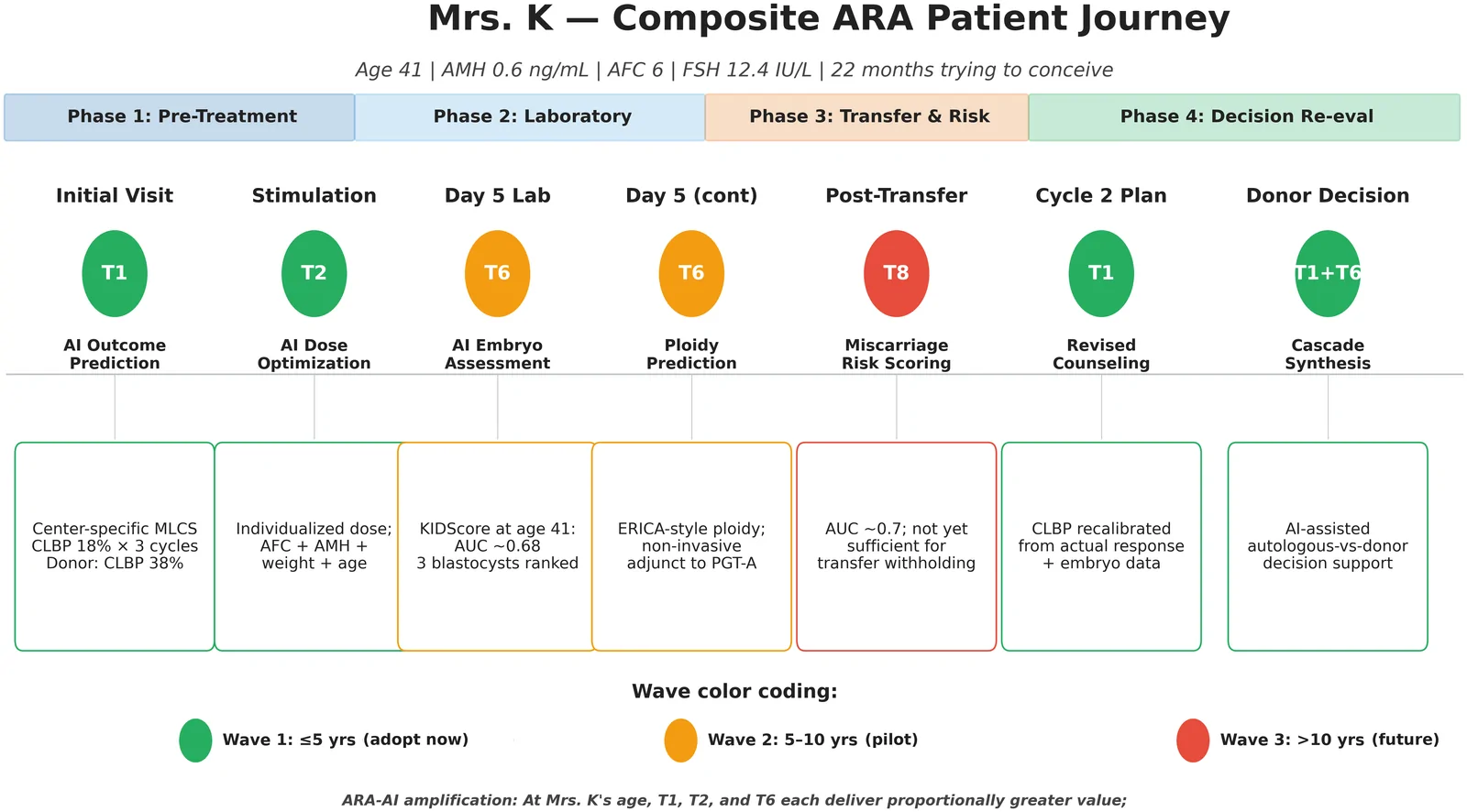
Clinical vignette — Mrs. K: composite advanced reproductive age (ARA) patient’s journey through the AI-integrated IVF protocol. Patient profile: age 41, AMH 0.6 ng/mL, AFC 6, FSH 12.4 IU/L, 22 months unsuccessful conception. Seven discrete technology touchpoints, plus a cross-cutting T3 RFID quality-tracking banner, are traced through four clinical phases: T1 (initial outcome prediction and revised counseling), T2 (stimulation dosing), T6 (embryo assessment via KIDScore; AUC 0.679 at age 41–42, 1), T6 (ploidy prediction using the ERICA algorithm, 77 — distinct from the T8 ERICA miscarriage application), T8 (miscarriage risk scoring; AUC ~0.70, not yet sufficient for transfer withholding), T1 (revised counseling from actual cycle response), and T1+T6 (cascade synthesis for autologous-versus-donor decision support). A horizontal T3 banner spanning all phases represents RFID electronic witnessing, which is active throughout the laboratory workflow and carries amplified medicolegal importance in ARA cases where each embryo is irreplaceable. Detail annotation cards below each touchpoint show clinical parameters and evidence metrics. Wave color coding: blue = Wave 1 (≤5 yr, Adopt Now); amber = Wave 2 (5–10 yr, Pilot); red = Wave 3 (>10 yr, Future). ARA-AI amplification T1, T2, and T6 each deliver proportionally greater discriminatory value at advanced reproductive age than in younger patient cohorts.

Mrs. K is a composite figure, drawn from the clinical features representative of advanced reproductive age patients with diminished ovarian reserve (**Figure 3**). The clinical scenario that follows describes care that could be delivered today, in 2026, for Mrs. K using only the Wave 1 and Wave 2 technologies in adequately resourced centers. T1 outcome prediction, T2 stimulation dosing, and T3 RFID quality tracking are deployable now, whereas the T6 AI embryo assessment is available adjunctively in time-lapse-equipped centers. While the T8 miscarriage risk prediction trajectory is beyond 2031 for routine deployment, it could inform the ARA-specific research agenda now. Each decision point in her care outlined below maps to a specific AI technology, a specific evidence finding, and a specific deployment status in 2026.

### Patient vignette: Mrs. K

Mrs. K is 41 years old, presenting after 22 months of unsuccessful conception. Her AMH is 0.6 ng/mL; antral follicle count is 6; day-3 FSH is 12.4 IU/L. She has completed two unsuccessful intrauterine insemination cycles and arrives at the clinic asking three questions: “Should we try IVF? What are my chances? And how should we plan if it doesn’t work?”

### Initial counseling: AI outcome prediction (T1)

For Mrs. K, prognosis counseling is the most consequential interaction with the clinic. Her age and ovarian reserve markers place her in a population where cumulative live birth probability (CLBP) estimates are miscalibrated against center-specific outcomes. A center-specific MLCS model trained on the clinic’s historical data (22) refines such estimates beyond national registry-based models. In the published MLCS validation, the model reclassified 23% of patients to CLBP ≥50% and 11% to CLBP ≥75%, correcting systematic prognosis underestimation—substantially more informative than population averages. These improvements translate directly to Mrs. K’s decision: whether to invest emotional and financial resources in three autologous attempts or consider donor oocytes earlier. This could be where AI delivers its highest-confidence near-term clinical value—software-only, EHR-integrated, and addressing prognosis miscalibration that affects every ARA patient.

### The stimulation cycle: AI dosing optimization (T2)

Mrs. K elects to proceed with two autologous cycles before reconsidering donor oocytes. For stimulation, the clinic uses an AI starting-dose model incorporating AMH, AFC, weight, age, and prior response when available. SHapley Additive exPlanations (SHAP) analysis of large IVF datasets identifies AFC and gonadotropin dosage among the top live birth predictors (100), and retrospective analyses of ML-guided dosing show reduced cycle cancellation and improved oocyte yield in low-prognosis patients (23, 32). For Mrs. K, individualized dose calibration matters more than for a 32-year-old with normal reserve because conventional age-adjusted protocols systematically under- or over-respond in this population (44). Emerging dynamic mid-cycle adjustment models will further refine the stimulation trajectory based on real-time follicular response.

### Embryo selection: AI assessment (T6)

Mrs. K’s first cycle yields three blastocysts. This is where the key ARA-AI amplification finding becomes operative. KIDScore AUC for live birth prediction rises from 0.596 at age <35 to 0.640 (35–37), 0.646 (38–40), 0.679 (41–42), and 0.768 at ≥43 (1). At Mrs. K’s age, the AI system’s discriminatory advantage over morphological assessment alone is at the upper end of its operative range, reflecting wider embryo-to-embryo quality variation from rising aneuploidy. The AI system can stratify implantation probability for the three blastocysts in a way morphological assessment cannot. While the 5–10 year horizon for T6 still applies to other subgroups, for ARA patients today, AI embryo assessment in time-lapse-equipped centers provides immediate adjunctive ranking value—CHLOE Blast or comparable platforms can provide explainable rankings communicated alongside conventional morphological grades.

### Ploidy prediction: T6 evolution toward non-invasive PGT-A

For Mrs. K, the aneuploidy rate among her blastocysts is expected to approach 70% at age 41, rising to >80% by age 43–45 (2, 101). PGT-A is the current gold standard but is invasive and requires biopsy expertise. AI models predicting ploidy from static images achieve ~70% accuracy (77); however, PGT-A interpretation itself remains contested following evidence that mosaic blastocysts can yield viable ongoing pregnancies (102), introducing additional complexity that future non-invasive AI ploidy models will need to incorporate; the parallel extension of embryo screening to polygenic risk raises distinct scientific and ethical questions that remain unresolved (103–105). While AI cannot yet replace PGT-A as a definitive selector, it can prioritize which blastocysts to biopsy first or provide adjunctive ploidy information—the embryo assessment frontier where ARA patients could benefit most between now and 2031.

### After transfer: AI miscarriage risk prediction (T8)

Mrs. K’s first euploid blastocyst is transferred and yields a positive pregnancy test. Yet first-trimester miscarriage rises sharply with maternal age, exceeding 40% at ≥40 versus 10–15% at <35 in general-population data (81), driven by age-related oocyte aneuploidy (106). Even if AI-based preimplantation miscarriage risk scoring (31) could identify which frozen blastocysts carry higher risk, the current AUC of ~0.70 is not sufficient for advising against transfer.

### Cycle 2 and beyond: T1, T2, T6 in cascade

Should Mrs. K’s autologous cycles fail, the same MLCS model that informed her initial counseling can generate a revised CLBP estimate from her actual stimulation response, oocyte yield, fertilization rate, and embryo cohort quality. AI-assisted donor decision support, expected to mature in the 5–10 year horizon, will guide the autologous-versus-donor decision more precisely. Mrs. K’s case illustrates a typical ARA cascade: T1 frames the conversation, T2 maximizes biological yield, T6 selects optimally from limited embryos, and T8 will eventually inform implantation prognosis. Each technology delivers proportionally greater value in her case than in a younger patient with more forgiving ovarian reserve.

### Why ARA patients set the implementation bar

Mrs. K’s journey illuminates why ARA patients could benefit most from AI integration. Technologies that deliver modest gains in younger patients could deliver disproportionately large gains here, driven by three converging amplifications: embryo quality variation widens with age, amplifying AI-based selection value (T6); individualized stimulation calibration matters more in diminished ovarian reserve (T2); and prognosis miscalibration carries higher impact when treatment windows are short (T1). The technology priority hierarchy for the ARA-focused 2026 clinic is therefore: T1 outcome prediction first, T2 stimulation dosing second, T6 embryo selection third (adjunctive in time-lapse-equipped centers), with T8 miscarriage risk prediction emerging as a Wave 3 priority. This exemplifies concentrated AI application to the IVF population for whom every decision matters.

## Discussion

This review evaluated nine candidate AI technologies across the IVF clinical workflow. It organizes them into three deployment waves and envisions their implications for ARA patients. The principal finding is that readiness for clinical deployment is asymmetric with respect to development profile and clinical anticipation: Wave 1 software-only tools are deployable now with minimal capital, while several aspirational technologies remain hardware- or ethics-constrained beyond 2031. The potential age-dependent amplification of AI performance suggests ARA patients as the population for whom these tools yield the greatest incremental value.

The three-wave adoption framework is the review’s principal contribution to clinical decision-making. Wave 1 technologies (T1–T3) share a defining characteristic absent from most AI-in-IVF discussions: they require no hardware capital expenditure — though EHR and LIMS integration carries substantial informatics labor costs. Wave 2 technologies (T4–T7) require multicenter prospective validation, and in most jurisdictions regulatory clearance. The divergent results from the iDAScore (27) and LOTUS (28) RCTs suggested that AI embryo selection is platform-specific, not a category-level technology. Efficacy data from one cleared platform cannot be extrapolated to another.

Although the nine technologies were validated in IVF, the underlying clinical problems — ovarian response, gamete quality, embryo competence, endometrial receptivity, outcome prediction — are shared across reproductive medicine. T2 stimulation dosing could be applied directly to intrauterine insemination (IUI) and fertility preservation, and AI-supported elective single embryo transfer has been proposed as a further extension (107); T7 endometrial receptivity is relevant to frozen embryo transfer and recurrent pregnancy loss; T1 outcome prediction can be applied to improve oncofertility and social egg freezing.

Furthermore, the ARA patient population emerges as the clearest priority for AI investment. The progressive increase in AI embryo selection AUC with maternal age (1) reflects that the wider embryo quality variation in older patients creates the discriminatory context in which AI pattern recognition outperforms human morphological assessment. This deviates from the conventional assumption that AI provides a uniform benefit. In this case, its value could be amplified the most in the population with the greatest clinical need.

As detailed in the per-technology evaluation (T6 section), the ARA-amplification thesis advanced throughout this review rests on a single retrospective platform-specific analysis (1) and remains a hypothesis-generating observation pending prospective age-stratified multicenter validation. This limitation does not alter the deployment-readiness analysis but does mean that the designation of ARA patients as the primary AI-beneficiary population must be regarded as provisional until prospective validation data accrue.

Beyond T1–T9, the 2031 horizon will be shaped by parallel developments such as AI-driven pharmacogenomics, oocyte cryopreservation outcome prediction, AI-microfluidic sperm selection, multi-omics integration (108, 109), and generative adversarial network (GAN)-generated synthetic data (110, 111) to overcome multicenter privacy constraints.

Several analytical gaps limit this review: inherent selection bias as a narrative review; NASSS assessment not validated against empirical adoption data; several technologies (T5b and T7) are resting on single-center or company-reported data without independent replication, with T4 partially exempt (FOLLISCAN has peer-reviewed multicenter validation [25], though CycleClarity remains company-reported); and the cited evidence base draws predominantly from high-income settings (United States, European Union, United Kingdom, Japan, Australia), with limited primary data from low- and middle-income countries (LMICs), constraining generalizability to global IVF practice and to centers operating under different regulatory, reimbursement, and digital-infrastructure conditions.

Important questions remaining for future research include, for example: whether live-birth-endpoint RCTs of AI embryo selection produce concordant results across platforms and populations; whether AI stimulation dosing translates the prospective evidence base into measurable reductions in ovarian hyperstimulation syndrome (OHSS) risk; whether the STAR system’s (microfluidic sperm recovery for non-obstructive azoospermia) first clinical pregnancy (68) can be replicated at scale in non-obstructive azoospermia cohorts; and whether AI miscarriage risk prediction reaches clinically actionable AUC levels (>0.80) after multi-stream data integration.

## Conclusion

The 2031 AI-integrated IVF clinic, as projected by this analysis, is a stratified reality. First, Wave 1 AI tools require no hardware capital and can integrate within 12–18 months, making outcome prediction (T1), stimulation dosing (T2), and RFID quality tracking (T3) the immediate near-term priorities. Second, AI embryo selection (T6) is available adjunctively now in time-lapse-equipped centers and will serve as the primary selection criterion within the decade; however, divergent RCT results indicate that AI embryo assessment is platform-specific and cannot be adopted as a category. Center-specific validation is required. Third, Wave 2 endometrial receptivity (T7) is a high-priority Wave 2 research target requiring prospective multicenter validation, while Wave 3 miscarriage prediction (T8) and robotic ICSI (T9) require foundational ethical and engineering development beyond 2031 before they can become routine practice. Among the remaining Wave 2 technologies, AI ultrasound monitoring (T4) and sperm analysis and selection (T5) require independent multicenter validation before clinical adoption can be recommended.

AI embryo selection is the highest-priority near-term target for ARA-focused programs, pending prospective age-stratified multicenter validation: KIDScore AUC has demonstrated steep age-dependent improvement (1) — meaning AI-based selection yields the greatest incremental value precisely where clinical need is the highest.

Between now and 2031, four key tasks are rate-limiting: multicenter RCT completion with live birth endpoints, regulatory pathway harmonization, ethical frameworks for AI-informed transfer decisions, and workforce training and IT data infrastructure. Whether the AI-integrated IVF clinic fulfills its promise depends not on algorithmic sophistication but on the rigor with which the field validates, governs, and integrates these technologies into routine practice. Delays in any one of these key tasks will push the 2031 vision toward 2033–2035.

For IVF clinic directors planning AI integration, the following implementation pathway emerges from this analysis. Months 1–6: deploy T1 (AI outcome prediction) for ARA patient counseling and donor-versus-autologous decision support; the software-only requirement and existing multicenter validation justify immediate adoption. Months 6–12: pilot T2 (AI stimulation dosing) in low-prognosis cohorts, with prospective tracking of MII oocyte yield as a primary metric; concurrently implement T3 (RFID quality tracking) as standard laboratory infrastructure. Year 2: evaluate T6 (AI embryo assessment) platforms specifically for ARA patients; for centers with time-lapse infrastructure, KIDScore’s AUC progression with maternal age (0.596 → 0.768) supports targeted adoption for this subpopulation. Year 3–5: pilot T4 (AI ultrasound monitoring; FOLLISCAN as the FDA-cleared peer-reviewed leader), T5 (sperm selection in research collaborations), and T7 (endometrial receptivity assessment) as capital infrastructure permits. Year 5+: monitor T8 (miscarriage risk prediction) for AUC improvements above the current ~0.70 threshold, and track T9 (robotic ICSI) for resolution of remote-procedure regulatory frameworks. This pathway reflects the central finding of this review: AI integration into IVF is not a single decision but a sequenced strategy, with immediate clinical value available in 2026 for clinics that prioritize software-first, evidence-validated deployment.

## Supplementary material

Supplementary Table 1 Five-year AI-IVF field progression milestones (2021–2026).

Supplementary Table 2 Combined technology assessment — evidence grading and viability.

Supplementary Table 3 Integrated IVF technology and clinical phase reference.

Supplementary Table 4 PRISMA-aligned search documentation.

Supplementary Table 5 Projected AI-integrated IVF workflow.

Supplementary Table 6 Resource impact analysis by technology domain.

## Glossary

AFC: antral follicle count
AI: artificial intelligence
AMH: anti-Müllerian hormone
ARA: advanced reproductive age
ART: assisted reproductive technology
ASRM: American Society for Reproductive Medicine
AUC: area under the receiver-operating curve
BMI: body mass index
CapEx: capital expenditure
CDRH: Center for Devices and Radiological Health
CE: Conformité Européenne
CGMH: Chang Gung Memorial Hospital
CI: confidence interval
CLBP: cumulative live birth probability
COI: conflict of interest
cpls: couples
CPR: clinical pregnancy rate
DB: double-blind
DOR: diminished ovarian reserve
EHR: electronic health record
ERA: endometrial receptivity assay
ESHRE: European Society of Human Reproduction and Embryology
ET: embryo transfer
EU: European Union
FDA: Food and Drug Administration
FET: frozen embryo transfer
FHIR: Fast Healthcare Interoperability Resources
FSH: follicle-stimulating hormone
FU: follow-up
GnRH: gonadotropin-releasing hormone
HEPES: N-2-hydroxyethylpiperazine-N-2-ethanesulfonic acid
HFEA: Human Fertilization and Embryology Authority
HL7: Health Level Seven
ICC: intraclass correlation coefficient
ICER: incremental cost-effectiveness ratio
ICSI: intracytoplasmic sperm injection
IDEAL: Idea, Development, Exploration, Assessment, Long-term
IMDRF: International Medical Device Regulators Forum
IUI: intrauterine insemination
IVF: *in vitro* fertilization
JARG: Journal of Assisted Reproduction and Genetics
KPI: key performance indicator
LB: live birth
LBP: live birth probability
LBR: live birth rate
LIMS: laboratory information management system
LLM: large language model
LOTUS: Live birth Outcomes from Time-lapse Using AI (trial name)
MC: multicenter
MDR: Medical Device Regulation (EU)
MHRA: Medicines and Healthcare products Regulatory Agency
MII: metaphase II (oocyte)
ML: machine learning
MLCS: machine learning center-specific
MLTRL: machine learning technology readiness level
NASSS: Non-adoption, Abandonment, Scale-up, Spread, and Sustainability
NI: non-inferiority
NMPA: National Medical Products Administration
NOA: non-obstructive azoospermia
NS: not significant
Obs: observational
OCEBM: Oxford Centre for Evidence-Based Medicine
OHSS: ovarian hyperstimulation syndrome
OpEx: operating expenditure
PCCP: Predetermined Change Control Plan
PGT-A: preimplantation genetic testing for aneuploidy
PMDA: Pharmaceuticals and Medical Devices Agency
PoC: proof-of-concept
PRISMA: Preferred Reporting Items for Systematic Reviews and Meta-Analyses
pts: patients
RCT: randomized controlled trial
RFID: radio-frequency identification
RIF: recurrent implantation failure
RPL: recurrent pregnancy loss
SaaS: Software-as-a-Service
SaMD: Software as a Medical Device
SART: Society for Assisted Reproductive Technology
SC: single-center
SLA: service-level agreement
STAR: Sperm Tracking and Recovery (system)
TCO: total cost of ownership
TFDA: Taiwan Food and Drug Administration
TGA: Therapeutic Goods Administration
TL: time-lapse
TRL: technology readiness level
UKCA: UK Conformity Assessed
WOI: window of implantation
XAI: explainable AI
