# Supplementary material for: Implementation readiness of artificial intelligence in in vitro fertilization: a multi-framework appraisal with special reference to advanced reproductive age patients
Source: Front Endocrinol (Lausanne). 2026 Sep 7;17:1910448. doi: 10.3389/fendo.2026.1910448 (PMC13591153; doi:10.3389/fendo.2026.1910448)
Supplement: Supplementary file 1 [file DataSheet1.pdf]

# Supplementary Methods

Supplementary information accompanying the manuscript:

*AI-Integrated IVF by 2031: Implementation Readiness and Potential Implications for Advanced-Age Patients* (Chang CL, 2026).

This document provides the full methodological detail abbreviated in the main manuscript Methodology section. Section numbering (S.M.1 through S.M.7) corresponds to the methodological elements referenced in the main text.

## S.M.1 Multi-Framework Approach

This review applies a multi-framework approach combining the Technology Readiness Level (TRL) and Machine Learning TRL (MLTRL) scales, the IDEAL framework for evaluating innovative interventions, Rogers' Diffusion of Innovation theory, and the NASSS framework for non-adoption and abandonment. Evidence is graded using an Oxford Centre for Evidence-Based Medicine (OCEBM)-adapted hierarchy. The nine candidate technologies span the full IVF protocol: outcome prediction, stimulation dosing, ultrasound monitoring, sperm and oocyte selection, embryo assessment (the highest-priority aspirational technology), endometrial receptivity assessment, miscarriage risk prediction, robotic ICSI, and RFID-based quality tracking. These nine technologies are mapped against four readiness dimensions and analyzed for the systematic amplification of AI benefit in advanced reproductive age (ARA) patients.

## S.M.2 Search Strategy and Inclusion Criteria

This narrative review searched PubMed/MEDLINE, Scopus, Google Scholar, and IEEE Xplore using AI and IVF-related terms (full database list, search strings, and inclusion logic in Supplementary Table S4). Coverage: English-language peer-reviewed articles, with a core search window of January 2021–March 2026 supplemented by foundational papers from January 2015 onward for technologies with pre-2021 origins, complemented by American Society for Reproductive Medicine (ASRM) and European Society of Human Reproduction and Embryology (ESHRE) conference programs and regulatory filings (FDA, EU Medical Device

Regulation [MDR]). Conference presentations are identified as such and not treated as equivalent to peer-reviewed evidence (Supplementary Table S1).

Non-peer-reviewed sources were included where they constituted the only available evidence for specific milestones, given the well-documented lag between commercial AI deployment and academic publication. These are classified at evidence Level 4–5 (case series, proof-of-concept, or expert opinion under the OCEBM hierarchy) in Supplementary Table S2. A total of 97 references are included in the final reference list, comprising peer-reviewed articles, regulatory documents, and conference proceedings. The systematic elements of the literature search are documented in Supplementary Table S4, which is Preferred Reporting Items for Systematic Reviews and Meta-Analyses (PRISMA)-aligned.

## S.M.3 Analytical Frameworks

The frameworks are complementary: TRL/MLTRL addresses technical maturity (Lavin et al., 2022); IDEAL and IDEAL-D address procedural staging (McCulloch et al., 2009; Marcus et al., 2022); Rogers' Diffusion of Innovation addresses adoption trajectory (Rogers, 2003); NASSS addresses organizational complexity not captured by evidence hierarchies alone (Greenhalgh et al., 2017). The DECIDE-AI reporting guideline informs early-stage clinical evaluation standards (Vasey et al., 2022).

### TRL/MLTRL Level Definitions

The nine levels of the TRL framework are conventionally grouped as follows: TRL 1–3 cover basic principles and proof-of-concept; TRL 4–6 cover component and system validation in laboratory and relevant environments; and TRL 7–9 cover system demonstration, qualification, and routine deployment in real-world settings. The MLTRL extension applies the same staging logic to machine learning systems, with explicit attention to data readiness, model robustness, and integration with operational infrastructure (Lavin et al., 2022). Across the nine technologies in this review, current positions range from TRL 4 (T7, T8) to TRL 8–9 (T3). The TRL assignments are summarized in Supplementary Table S2.

## S.M.4 NASSS Domain Complexity Assessment

Table 1 in the main manuscript presents the NASSS domain complexity heatmap (light blue = Simple, amber = Complicated, dark navy = Complex). Technologies rated X in more than three domains are unlikely to achieve mainstream adoption without fundamental resolution (Greenhalgh et al., 2017). RFID quality tracking is the only technology rated Simple overall; robotic ICSI and miscarriage prediction carry X ratings in four or more domains. The Wider System domain (regulatory, economic, and political environment) rates Complicated or Complex for six of nine technologies, reflecting immature AI-specific regulatory frameworks (European Commission, 2024).

## S.M.5 Structured Sensitivity Analysis of NASSS Ratings

To assess the robustness of the NASSS ratings, a structured re-rating exercise was conducted in which the original assignments were independently re-evaluated against their evidence sources. Overall agreement was 87% (55/63 domains). Four adjudicated ratings all moved toward higher complexity, summarized below.

- Robotic ICSI – Technology domain (C→X): fundamental speed constraint.
- Miscarriage Prediction – Adopter System domain (C→X): ethical objections to autonomous transfer decisions.
- Endometrial Receptivity AI – Wider System domain (S→C): Software as a Medical Device (SaMD) regulatory uncertainty across multiple jurisdictions.
- Sperm Selection – Value Proposition domain (S→C): surrogate endpoint dependence in all trials (Carrión-Sisternas et al., 2025).

Full NASSS-domain ratings, including pre- and post-adjudication assignments for each of the nine technologies across seven domains, are tabulated in Supplementary Table S2.

## S.M.6 Limitations

As a narrative review, selection bias is inherent. Framework assessments involve author judgment and are not validated against empirical IVF AI adoption data. Commercial products are cited as illustrative examples, not endorsements. Several key studies were conducted by investigators affiliated with the companies whose products they evaluate; these conflicts are addressed in the COI screening methodology below.

## S.M.7 Conflict of Interest Screening Methodology

A systematic conflict-of-interest (COI) assessment was integrated into evidence evaluation. Author affiliations and funding sources were cross-referenced against developers of evaluated algorithms. Studies where authors held direct employment or advisory roles with the developer were flagged. These studies received higher risk-of-bias ratings in Table 2 (main manuscript) because they often constitute the only available data for early-stage tools.

Specific COI-related evidence classifications include the following:

- The Shoham (2025) Fairtility/CHLOE Blast 510(k) reference is a company press release, classified at evidence Level 5.
- T4 ultrasound monitoring has two FDA-cleared platforms: CycleClarity (2026; company-reported data, classified at Level 4) and FOLLISCAN (MIM Fertility; peer-reviewed multicenter validation by Wygocki et al., 2026 in JARG).
- Both Level 4–5 references are treated as directional benchmarks rather than definitive evidence.
