# Supplementary material for: Implementation readiness of artificial intelligence in in vitro fertilization: a multi-framework appraisal with special reference to advanced reproductive age patients
Source: Front Endocrinol (Lausanne). 2026 Sep 7;17:1910448. doi: 10.3389/fendo.2026.1910448 (PMC13591153; doi:10.3389/fendo.2026.1910448)
Supplement: Supplementary file 2 [file Table1.docx]

# Supplementary Table Legends

**Supplementary Table S1. Five-Year AI-IVF Field Progression Milestones (2021–2026).** Five field-level milestones comparing status in ~2021 versus 2026: regulatory clearances for AI embryo tools, completed randomized controlled trials, robotic ICSI feasibility, global AI adoption rates, and validation framework consensus. Together these provide the empirical basis for 2031 projections.

**Supplementary Table S2. Combined Technology Assessment — Evidence Grading and Viability.** Nine AI technologies (T1–T9) assessed across six dimensions: OCEBM evidence level, TRL, scale potential, ARA patient benefit, literature depth (number of key peer-reviewed studies and regulatory documents directly supporting the technology’s clinical claims), and key limitation/concern. This represents a best-case scenario; actual implementation varies by clinic size, geography, and regulatory environment.

**Supplementary Table S3. Integrated IVF Technology and Clinical Phase Reference.** Nine AI technologies (T1–T9) organized by clinical phase (Phase 1 Pre-Treatment, Phase 2 Laboratory, Phase 3 Transfer, Phase 4 Governance), clinical scope, and deployment readiness tier (≤5 yr, 5–10 yr, >10 yr).

**Supplementary Table S4. PRISMA-Aligned Search Documentation.** Database sources, date ranges, primary search strings, and article counts for the systematic literature search. Four databases/sources were searched: PubMed/MEDLINE (January 2015 – March 2026), Scopus, Google Scholar, IEEE Xplore, and ASRM/ESHRE conference programs supplemented by FDA and EU MDR regulatory filings. Search terms combined AI/ML/deep learning terminology with IVF/ART/embryo selection terms. The search is aligned with PRISMA reporting standards for transparency and reproducibility.

**Supplementary Table S5. Projected AI-Integrated IVF Workflow.** Ten AI technology entries (T1–T9, with T6 split into T6 Embryo Assessment and T6* Genetic Testing) listed by T-number. Columns: technology category, expected clinical benefit with supporting metrics, and key references. Technologies span four clinical phases: Pre-Treatment (T1, T2, T4), Laboratory (T5, T6, T6*, T9), Transfer (T7, T8), and Governance (T3).

**Supplementary Table S6. Resource Impact Analysis by Technology Domain.** Four dimensions rated across nine AI technologies (T1–T9): time savings, cost savings, training impact, and clinical quality/standardization. Impact magnitude: ●●● = High, ●●○ = Moderate, ●○○ = Low/Uncertain. Each rating followed by supporting clinical justification.

# Supplementary Tables

**Supplementary Table S1. Five-Year AI-IVF Field Progression Milestones (2021–2026).**

| **Milestone** | **Status ~2021** | **Status 2026** |
| --- | --- | --- |
| Regulatory clearance for AI embryo tools | No AI embryo assessment tool cleared by any regulatory body worldwide | 510(k) clearance granted (Sep 2025); EU CE marking (Oct 2025) |
| Randomized controlled trials | No completed RCTs; debate over RCT appropriateness for AI validation | Two landmark RCTs completed and published (iDAScore NI failed; LOTUS NI met) |
| Robotic ICSI | Theoretical concept; no human application reported | ~20 cumulative live births (company-reported)(Mendizabal-Ruiz et al., 2025); commercial platform deployed (Memorial Hospital Istanbul, July 2025) |
| Global AI adoption rate | 24.8% of IVF professionals testing AI | 29.2% (AI integrated into IVF clinical workflow) adoption; 83.62% planning implementation within 1–5 years (Shoham G et al., 2025; same survey instrument as the 53.2% "any-use" figure cited in main text, narrower denominator) |
| Validation framework | No consensus framework for AI in reproductive medicine | "Croatia Consensus" proposed and under active development |

**Supplementary Table S2. Combined Technology Assessment — Evidence Grading and Viability.**

| **Tech** | **Evidence Level (OCEBM)** | **TRL** | **Scale Potential** | **ARA Patient Benefit** | **Literature Depth** | **Key Limitation / Concern** |
| --- | --- | --- | --- | --- | --- | --- |
| **T1** | Level 2 | TRL 7–8 | HIGH (software-only) | HIGH — age-specific LBR modelling | 5+ (Yao, Bulletti, Sadegh-Zadeh, Borji) | Retrospective multicenter; no prospective decision-impact study |
| **T2** | Level 3 | TRL 6 | HIGH (software-only) | HIGH — poor responder dosing optimization | 4+ retrospective studies (Correa 2022, Fanton 2022, Ortiz 2025, Letterie 2020/2022); no prospective RCT data | Prospective data unavailable; IDoser RCT (Correa et al., 2024 protocol) withdrawn before enrollment |
| **T3** | Level 2 | TRL 8–9 | HIGH (mature technology) | LOW — quality not age-specific outcome | 3 (Bori, Hanassab, Maggiulli) | Mature technology; limited IVF-specific RCT data; Age-neutral; no outcome benefit demonstrated |
| **T4** | Level 3 | TRL 7–8 | HIGH (software overlay) | LOW — efficiency, not outcome | 3 (FOLLISCAN [Wygocki et al., 2026, peer-reviewed multicenter]; CycleClarity [FDA-cleared, company-reported]; Letterie) | CycleClarity company-reported; FOLLISCAN has peer-reviewed multicenter validation |
| **T5** | Level 3–4 | TRL 5 | MODERATE (needs TL/hardware) | LOW — male factor, not ARA-specific | 4 (Carrión-S., Suryawanshi, Javadi, Riordon) | Single center; surrogate endpoint; no live birth data |
| **T6** | Level 1 | TRL 7–8 | HIGH (software) | MODERATE — aneuploidy key issue in ARA | 10+ (2 RCTs, multiple retrospective cohorts) | Two RCTs with divergent results; platform-dependent performance |
| **T7** | Level 3 | TRL 4 | MODERATE (software) | MODERATE — RIF patients benefit | 4 (Bornaun, Asch Schuff, Cheredath, Wang) | Single-center retrospective; synthetic data augmentation; no prospective multicenter |
| **T8** | Level 3 | TRL 4 | MODERATE (software) | MODERATE — age + miscarriage risk correlation | 4 (Amitai, Chavez-B., Wang, Liu) | Modest AUC ~0.7 at preimplantation stage; multifactorial etiology limits single-model |
| **T9** | Level 4 | TRL 5–6 | LOW (expensive HW, 4× slow) | LOW — not age-specific | 3 (Conceivable, Chavez-B., Mendizabal-R.) | Proof-of-concept only; 4× speed limitation vs. manual; hardware engineering constraint; all from overlapping groups |

**Supplementary Table S3. Integrated IVF Technology and Clinical Phase Reference.**

| **Phase** | **Clinical Scope** | **T#** | **Technology** | **Deployment Tier** |
| --- | --- | --- | --- | --- |
| **Phase 1 Pre-Treatment** | Patient counseling, stimulation planning, cycle monitoring | **T1** | AI Outcome Prediction | *≤5 years* |
|  |  | **T2** | AI Stimulation Dosing | *≤5 years* |
|  |  | **T4** | AI Ultrasound Monitoring | *5–10 years* |
| **Phase 2 Laboratory** | Gamete selection, fertilization, embryo culture and assessment | **T5** | Sperm AI | *5–10 years* |
|  |  | **T6** | AI Embryo Assessment and Selection | *5–10 years* |
|  |  | **T9** | Robotic ICSI | *>10 years* |
| **Phase 3 Transfer** | Endometrial readiness assessment, post-transfer surveillance | **T7** | AI Endometrial Receptivity Assessment | *5–10 years* |
|  |  | **T8** | AI Miscarriage Risk Prediction | *>10 years* |
| **Phase 4 Governance** | Sample tracking, laboratory quality, documentation | **T3** | RFID Sample Tracking and Quality Management | *≤5 years* |

**Supplementary Table S4. PRISMA-Aligned Search Documentation.**

| **Database / Source** | **Date Range Searched** | **Primary Search String** | **Articles Identified** |
| --- | --- | --- | --- |
| PubMed / MEDLINE | Jan 2015 – Mar 2026 | ("artificial intelligence" OR "machine learning" OR "deep learning") AND ("in vitro fertilization" OR "IVF" OR "assisted reproductive technology" OR "embryo selection") | 312 |
| Scopus | Jan 2022 – Mar 2026 | Same primary string; secondary: ("NASSS" OR "technology readiness") AND ("digital health" OR "fertility") | 184 |
| Web of Science | Jan 2022 – Mar 2026 | Same primary string; additional MeSH: "Fertilization in Vitro", "Artificial Intelligence" | 147 |
| Google Scholar | Jan 2022 – Mar 2026 | Same terms; top 100 results by relevance assessed | 100 |
| IEEE Xplore | Jan 2020 – Mar 2026 | "IVF" AND ("deep learning" OR "convolutional neural network" OR "robotics") | 68 |
| FDA 510(k) / EU MDR / TFDA | Jan 2023 – Mar 2026 | Manual search: "embryo", "IVF", "assisted reproduction" in device databases | 14 |
| Conference program | 2022–2026 | Full program documents; oral and poster abstracts reviewed | 46 abstracts |
| Backward/forward citation search | Continuous | Reference lists of all included reviews; forward citation search via Google Scholar | ≥50 |
| Total identified (after deduplication) | – | – | ~1,420 |

**Supplementary Table S5. Projected AI-Integrated IVF Workflow.**

| **Stage (T#)** | **Technology Category** | **Expected Benefit** | **Key Refs** |
| --- | --- | --- | --- |
| T1 Outcome Prediction | ML center-specific live birth prediction; LLM counseling support | Decision support; personalized counseling | (Chambers et al., 2009; Gély et al., 2026; Cao et al., 2025; Dinç et al., 2026; Yao et al., 2025; Borji et al., 2025) |
| T2 Stimulation Planning | ML-based FSH dose optimization and trigger timing prediction | Reduced trial-and-error dosing; FSH savings | (Correa et al., 2022; Fanton et al., 2022; Letterie et al., 2020, 2022; Ortiz et al., 2025) |
| T3 Quality Tracking | RFID/barcode witnessing; AI-automated KPI generation | Near-zero ID error; cost −25%, efficiency +35% | (Maggiulli et al., 2024; AI Fertility Society, 2026) |
| T4 AI Ultrasound Monitoring | AI-automated ultrasound follicle counting and measurement | 66% scan time reduction; standardization | (CycleClarity, 2026) |
| T5 Sperm AI | AI real-time sperm morphology and motility analysis | Blastocyst rate +10% (surrogate endpoint) | (Carrión-Sisternas et al., 2025; Curchoe et al., 2023) |
| T6 Embryo Assessment | Multi-modal fusion (time-lapse + deep learning) | Non-inferior to expert (RCT evidence) | (Zaninovic and Rosenwaks, 2020; Bormann et al., 2020; Sun et al., 2024; Kato et al., 2021) |
| T6* Genetic Testing | AI-enhanced non-invasive PGT-A (aneuploidy + PGT-P; Treff et al., 2020; Turley et al., 2021; Siermann et al., 2024) | Reduced biopsy-related damage; aneuploidy risk scoring | (Franasiak et al., 2014; Treff et al., 2020; Turley et al., 2021; Siermann et al., 2024; Horta et al., 2025) |
| T7 Endometrial Assessment | AI endometrial receptivity scoring; WOI prediction | Composite AUC 0.94 (single-center retrospective with synthetic augmentation; High RoB); reduced implantation failure | (Díaz-Gimeno et al., 2011; Asch Schuff et al., 2024; Bornaun, 2026; Liu et al., 2026) |
| T8 Miscarriage Prediction | AI morphodynamic/morphometric risk scoring from embryo images | AUC ~0.7; reduced emotional harm from unexpected miscarriage | (Magnus et al., 2019; Amitai et al., 2023; Chavez-Badiola et al., 2024; Liu et al., 2025) |
| T9 Robotic ICSI | Robotic ICSI; automated oocyte handling | Remote capability; standardized handling | (Chavez-Badiola et al., 2026; Mendizabal-Ruiz et al., 2025; Curchoe et al., 2023) |

**Supplementary Table S6. Resource Impact Analysis by Technology Domain.**

| **Technology Area** | **Time Savings** | **Cost Savings** | **Training Impact** | **Clinical Quality / Standardization** |
| --- | --- | --- | --- | --- |
| T1 Outcome Prediction | **●●●** Informed decisions faster | **●●●** futile cycles avoided | **●●●** Decision support integration | **●●●** Personalized counseling |
| T2 AI Stimulation Dosing | **●●○** Fewer monitoring visits | **●●○** FSH −20%; fewer cycles | **●●○** Novice → expert dosing | **●●●** Reduced inter-physician variability |
| T3 RFID/Quality Tracking | **●●●** Auto documentation | **●●●** −25% lab cost | **●●○** Reduces manual tracking | **●●●** Zero-error traceability |
| T4 AI Ultrasound Monitoring | **●●●** 66% scan reduction | **●●○** Reduced sonographer time | **●●○** Automated counting | **●●●** Standardized measurements |
| T5 Sperm AI | **●●○** Faster than manual | **●●○** Blastocyst rate +10% | **●●●** Objective scoring | **●●●** Reduced operator variability |
| T6 AI Embryo Selection | **●●○** Faster assessment | **●●○** Fewer failed transfers | **●●○** Reduces subjectivity | **●●○** Non-inferior (RCT evidence) |
| T7 Endometrial AI | **●●○** Fewer failed transfers | **●●●** avoided failure | **●●○** Automated scoring | **●●●** AUC 0.94 composite |
| T8 Miscarriage Prediction | **●●○** Avoids miscarriages | **●●○** avoided miscarriage | **●○○** No extra training needed | **●●○** Novel safety dimension |
| T9 Robotic ICSI | **●○○** 8.7 vs 2 min/oocyte | **●○○** High equipment cost | **●●●** Remote capability | **●●○** Reproducible technique |
